# Supplementary material for: Interaction of identity and beliefs with genetic literacy
Source: Am J Hum Genet. 2025 Dec 22;113(1):16–28. doi: 10.1016/j.ajhg.2025.11.014 (PMC12817316; doi:10.1016/j.ajhg.2025.11.014)
Supplement: Document S1. Figures S1–S5 and Tables S1–S18 [file mmc1.pdf]

The American Journal of Human Genetics, Volume *113*

Supplemental information

Interaction of identity and beliefs with genetic literacy

Gabriela M. Ramírez Renta, India D. Little, Laura M. Koehly, Anna J. Hilliard, Kaylee L. Foor, Jessica Butts, Jordan Lundeen, and Chris Gunter

Supplemental Figures and Legends

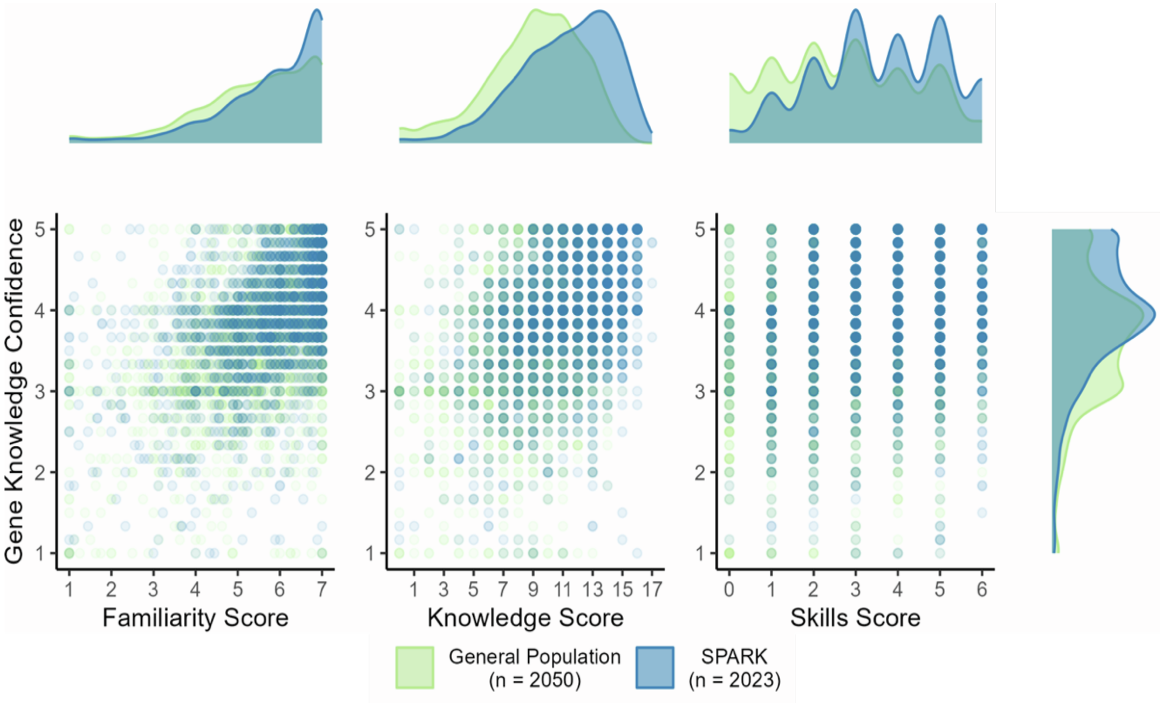

Figure S1: Scatter plots of genetic knowledge confidence by each genetic literacy score and population. Density plots of each genetic literacy score by population are provided in the upper margin. The density plot of the genetic knowledge confidence by population is provided in the right margin.

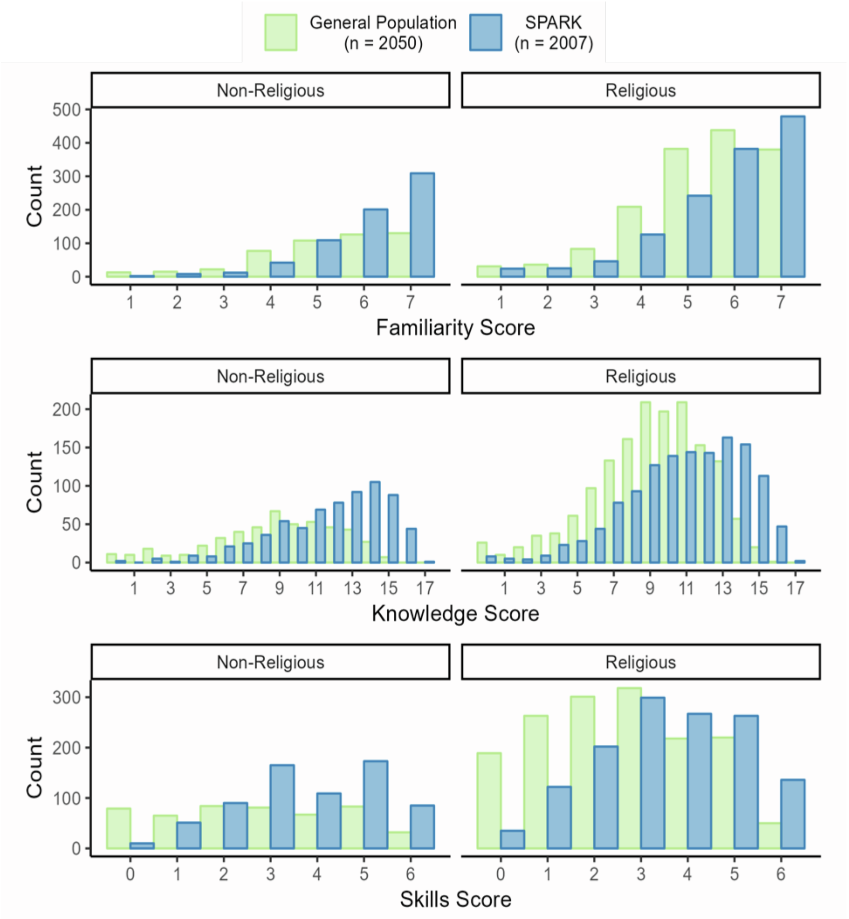

Figure S2: Histogram plots of each genetic literacy score by religious affiliation (Religious vs. Non-Religious) and population.

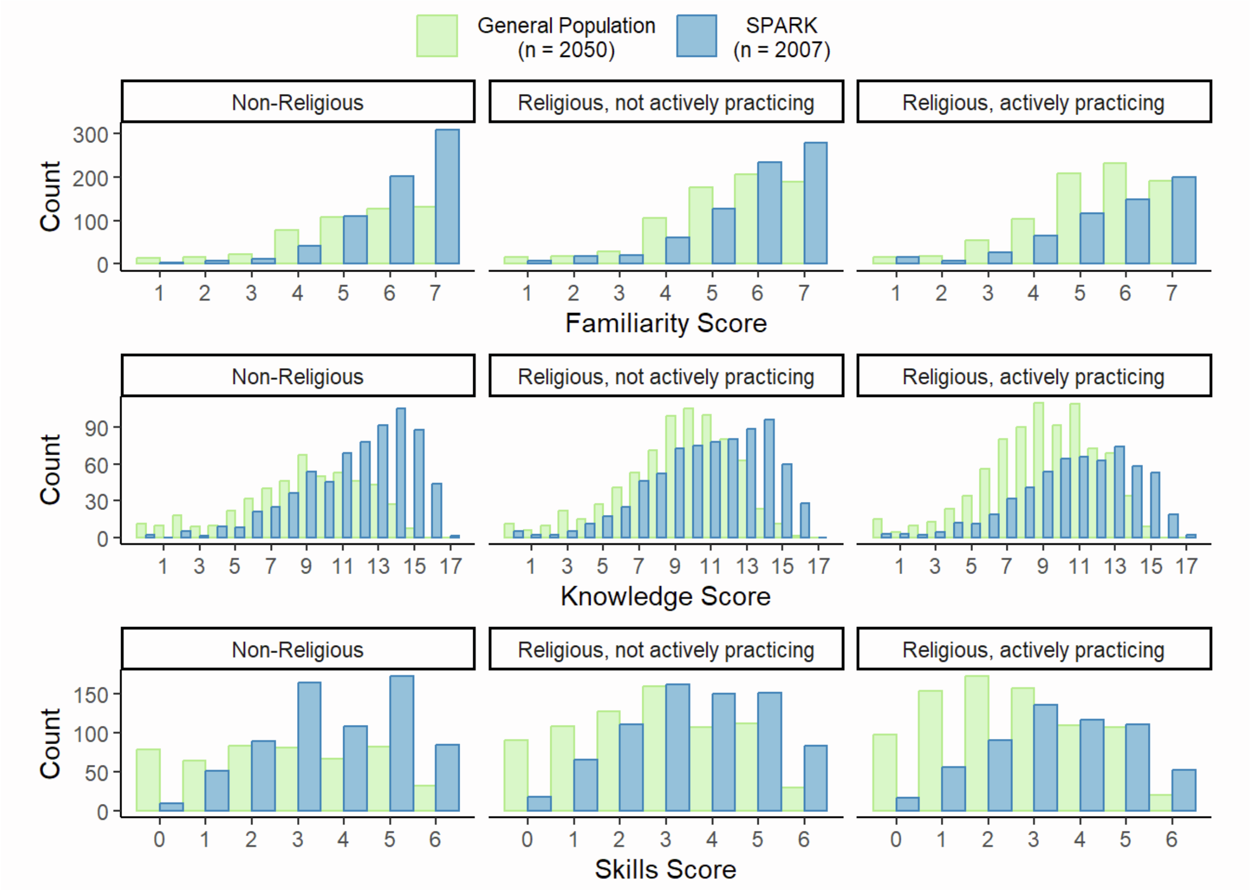

Figure S3: Histogram plots of each genetic literacy score by religiosity (Non-Religious vs. Religious, not actively practicing vs. Religious, actively practicing) and population.

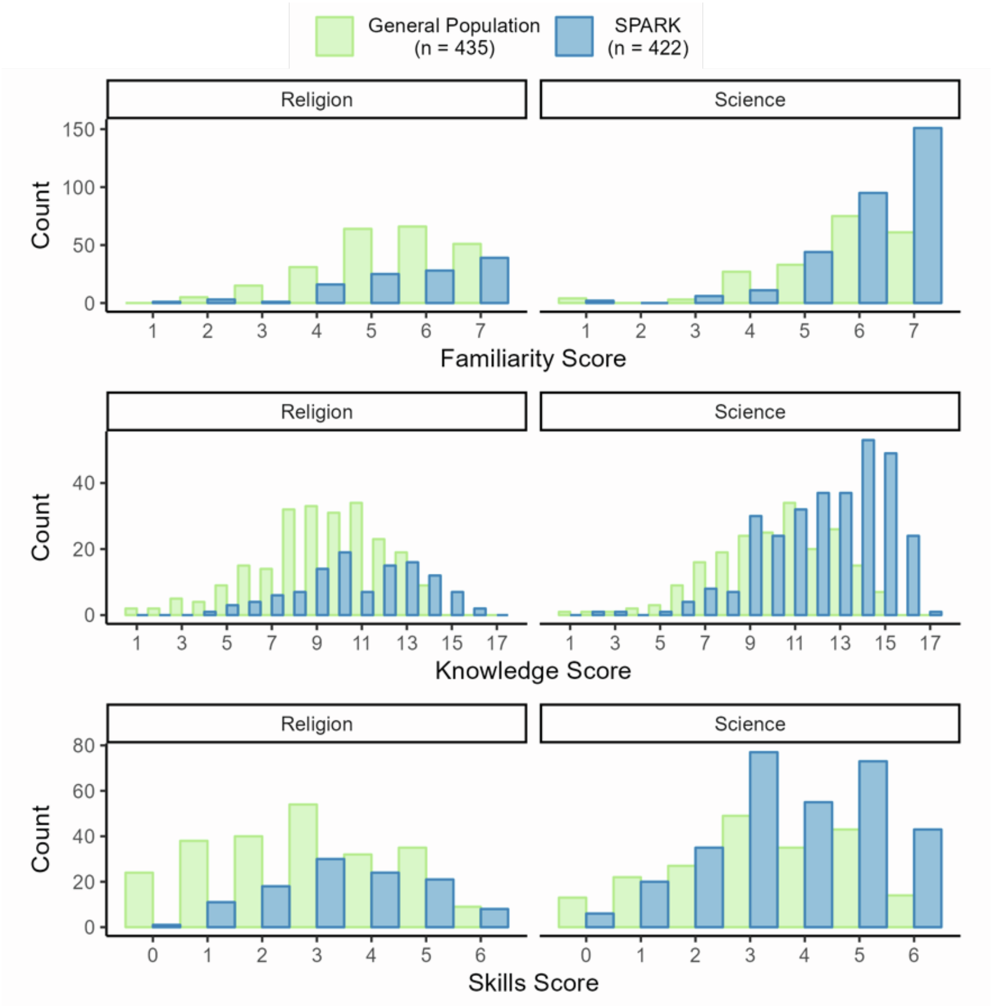

Figure S4. Histogram plots of each genetic literacy score by belief in Religion or Science (Religion vs. Science) and population.

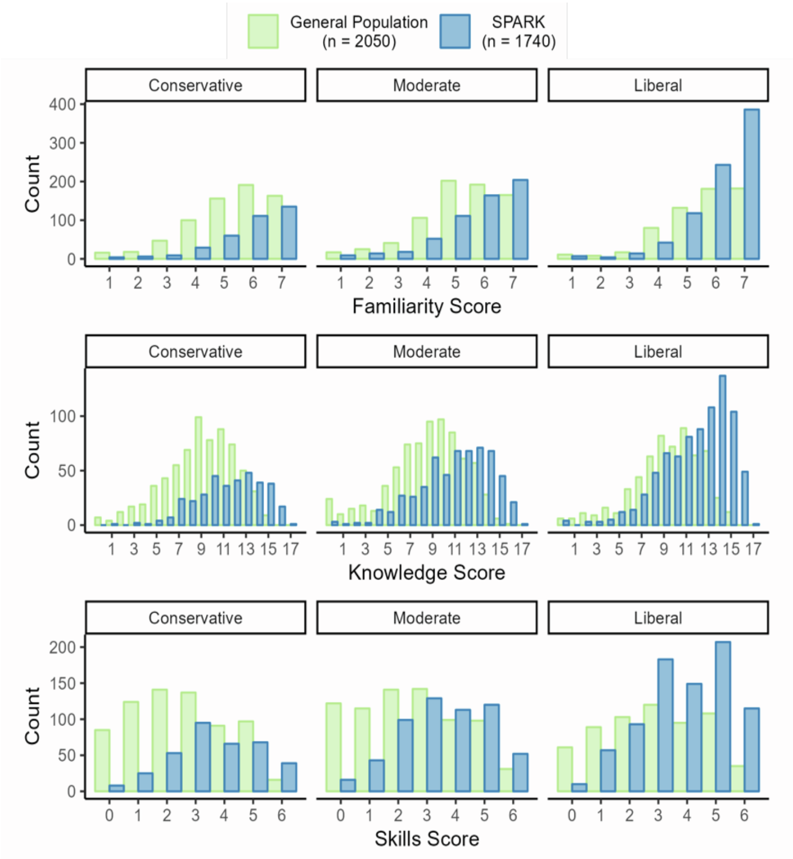

Figure S5. Histogram plots of each genetic literacy score by political belief and population.

Supplemental Tables

All tables showing linear regression models are formatted similarly: Each cell contains the unstandardized regression coefficient (standard error in parentheses), representing the change in GL subscale score associated with a one-unit increase in continuous predictors or the difference from the reference category for categorical predictors. R^2 indicates the proportion of variance in familiarity scores explained by each model. *n* = 4,057 unless otherwise indicated.

*** $p < 0.001$; ** $p < 0.01$; * $p < 0.05$.

	**Familiarity Score**	**Knowledge Score**	**Skills Score**
**Intercept**	3.508 *** (0.122)<br>p: <0.001	5.901 *** (0.297)<br>p: <0.001	1.364 *** (0.155)<br>p: <0.001
**Population - SPARK**	0.234 *** (0.041)<br>p: <0.001	1.856 *** (0.099)<br>p: <0.001	0.813 *** (0.052)<br>p: <0.001
**Education - High School**	0.615 *** (0.108)<br>p: <0.001	1.012 *** (0.262)<br>p: <0.001	0.556 *** (0.137)<br>p: <0.001
**Education - Undergraduate**	0.833 *** (0.112)<br>p: <0.001	1.532 *** (0.272)<br>p: <0.001	0.907 *** (0.142)<br>p: <0.001
**Education - Graduate**	1.069 *** (0.106)<br>p: <0.001	2.517 *** (0.258)<br>p: <0.001	1.102 *** (0.135)<br>p: <0.001
**Perceived Importance Genetic Knowledge**	0.196 *** (0.013)<br>p: <0.001	0.265 *** (0.031)<br>p: <0.001	0.085 *** (0.016)<br>p: <0.001
n	4057	4057	4057
R^2	0.114	0.166	0.108
Overall *F* statistic	104.056	161.305	98.235
Overall *F p* value	<0.001	<0.001	<0.001

*** $p < 0.001$; ** $p < 0.01$; * $p < 0.05$.

Table S1: Linear regression model results for each GL score by population, education, and perceived gene importance. Entries are the “coefficient (standard error),” and stars indicate statistical significance.

	Familiarity Score	Knowledge Score	Skills Score
**Intercept**	2.094 *** (0.12)<br>p: <0.001	2.77 *** (0.301)<br>p: <0.001	0.839 *** (0.165)<br>p: <0.001
**Population - SPARK**	0.12 ** (0.037)<br>p: 0.001	1.589 *** (0.094)<br>p: <0.001	0.771 *** (0.051)<br>p: <0.001
**Education - High School**	0.412 *** (0.099)<br>p: <0.001	0.613 * (0.248)<br>p: 0.013	0.477 *** (0.136)<br>p: <0.001
**Education - Undergraduate**	0.612 *** (0.103)<br>p: <0.001	1.087 *** (0.258)<br>p: <0.001	0.822 *** (0.141)<br>p: <0.001
**Education - Graduate**	0.749 *** (0.098)<br>p: <0.001	1.882 *** (0.246)<br>p: <0.001	0.978 *** (0.135)<br>p: <0.001
**Confidence in Genetic Knowledge**	0.753 *** (0.023)<br>p: <0.001	1.396 *** (0.058)<br>p: <0.001	0.299 *** (0.032)<br>p: <0.001
n	4057	4057	4057
R^2	0.255	0.256	0.121
Overall *F* statistic	277.333	279.426	111.647
Overall *F p* value	<0.001	<0.001	<0.001

*** $p < 0.001$; ** $p < 0.01$; * $p < 0.05$.

Table S2: Linear regression model results for each GL score by population, education, and genetic knowledge confidence. Entries are the “coefficient (standard error),” and stars indicate statistical significance.

	Familiarity Score	Knowledge Score	Skills Score
**Intercept**	4.652 *** (0.109)<br>p: <0.001	7.566 *** (0.26)<br>p: <0.001	1.951 *** (0.135)<br>p: <0.001
**Population - SPARK**	0.32 *** (0.041)<br>p: <0.001	1.952 *** (0.099)<br>p: <0.001	0.834 *** (0.051)<br>p: <0.001
**Education - High School**	0.679 *** (0.111)<br>p: <0.001	1.116 *** (0.264)<br>p: <0.001	0.598 *** (0.137)<br>p: <0.001
**Education - Undergraduate**	0.936 *** (0.115)<br>p: <0.001	1.701 *** (0.274)<br>p: <0.001	0.975 *** (0.142)<br>p: <0.001
**Education – Graduate**	1.184 *** (0.109)<br>p: <0.001	2.703 *** (0.26)<br>p: <0.001	1.175 *** (0.135)<br>p: <0.001
**Religious**	-0.210 *** (0.046)<br>p: <0.001	-0.472 *** (0.109)<br>p: <0.001	-0.238 *** (0.056)<br>p: <0.001
n	4057	4057	4057
R^2	0.068	0.155	0.106
Overall *F* statistic	59.563	148.962	96.231
Overall *F* *p* value	<0.001	<0.001	<0.001

*** $p < 0.001$; ** $p < 0.01$; * $p < 0.05$.

Table S3: Linear regression model results for each GL score by population, education, and religious affiliation. Entries are the “coefficient (standard error),” and stars indicate statistical significance.

	Familiarity Score	Knowledge Score	Skills Score
**Intercept**	4.656 *** (0.109)<br>p: <0.001	7.571 *** (0.26)<br>p: <0.001	1.956 *** (0.135)<br>p: <0.001
**Population - SPARK**	0.308 *** (0.041)<br>p: <0.001	1.936 *** (0.099)<br>p: <0.001	0.819 *** (0.051)<br>p: <0.001
**Education - High School**	0.674 *** (0.111)<br>p: <0.001	1.110 *** (0.264)<br>p: <0.001	0.592 *** (0.137)<br>p: <0.001
**Education - Undergraduate**	0.937 *** (0.115)<br>p: <0.001	1.702 *** (0.274)<br>p: <0.001	0.976 *** (0.142)<br>p: <0.001
**Education – Graduate**	1.195 *** (0.109)<br>p: <0.001	2.717 *** (0.26)<br>p: <0.001	1.1789 *** (0.135)<br>p: <0.001
**Religious, not actively practicing**	-0.125 * (0.051)<br>p: 0.015	-0.363 ** (0.122)<br>p: 0.003	-0.130 * (0.04)<br>p: 0.04
**Religious, actively practicing**	-0.306 *** (0.052)<br>p: <0.001	-0.593 *** (0.125)<br>p: <0.001	-0.358 *** (0.065)<br>p: <0.001
n	4057	4057	4057
R^2	0.072	0.156	0.109
Overall *F* statistic	52.092	124.871	82.827
Overall *F p* value	<0.001	<0.001	<0.001

*** $p < 0.001$; ** $p < 0.01$; * $p < 0.05$.

Table S4: Linear regression model results for each GL score by population, education, and religiosity. Beliefs are regressed against “Not religious.” Entries are the “coefficient (standard error),” and stars indicate statistical significance.

	Familiarity Score	Knowledge Score	Skills Score
**Intercept**	4.407 *** (0.11)<br>p: <0.001	6.963 *** (0.263)<br>p: <0.001	1.71 *** (0.138)<br>p: <0.001
**Population - SPARK**	0.374 *** (0.043)<br>p: <0.001	2.151 *** (0.102)<br>p: <0.001	0.884 *** (0.054)<br>p: <0.001
**Education - High School**	0.703 *** (0.114)<br>p: <0.001	1.151 *** (0.271)<br>p: <0.001	0.622 *** (0.143)<br>p: <0.001
**Education - Undergraduate**	0.933 *** (0.118)<br>p: <0.001	1.663 *** (0.281)<br>p: <0.001	0.925 *** (0.148)<br>p: <0.001
**Education - Graduate**	1.132 *** (0.112)<br>p: <0.001	2.533 *** (0.267)<br>p: <0.001	1.126 *** (0.14)<br>p: <0.001
**Political Belief - Conservative**	0.031 (0.053)<br>p: 0.561	0.329 ** (0.127)<br>p: 0.01	-0.016 (0.067)<br>p: 0.812
**Political Belief - Liberal**	0.301 *** (0.05)<br>p: <0.001	0.68 *** (0.118)<br>p: <0.001	0.295 *** (0.062)<br>p: <0.001
n	3790	3790	3790
R^2	0.083	0.178	0.12
Overall *F* statistic	57.166	136.742	86.21
Overall *F* *p* value	<0.001	<0.001	<0.001

*** $p < 0.001$; ** $p < 0.01$; * $p < 0.05$.

Table S5: Linear regression model results for each GL score by population, education, and political belief. Beliefs are regressed against “Political belief - Moderate.” Entries are the “coefficient (standard error),” and stars indicate statistical significance.

Tukey Comparisons of Means for Political Belief

	Familiarity Score	Knowledge Score	Skills Score
**Conservative – Moderate**	0.031 (0.05)<br>p: 0.830	0.329 * (0.13)<br>p: 0.026	-0.016 (0.07)<br>p: 0.969
**Liberal – Moderate**	0.301 *** (0.05)<br>p: <0.0001	0.680 *** (0.12)<br>p: <0.001	0.294 *** (0.06)<br>p: <0.00001
**Liberal – Conservative**	0.270 *** (0.05)<br>p: <0.0001	0.351 * (0.13)<br>p: 0.016	0.311 *** (0.07)<br>p: <0.00001

Table S6: Tukey pairwise comparison of means for each GL score by population, education, and political belief. Entries are the "coefficient (standard error)" and stars equal indicate statistical significance (*** $p < 0.001$; ** $p < 0.01$; * $p < 0.05$).

Interactions of Religious Affiliation, Religiosity, and Political Belief

We are unable to include both religious affiliation and religiosity in the same model because these two variables have overlapping definitions for “Non-Religious,” so we looked at religiosity and political belief and then religious affiliation and political belief separately.

Religiosity and Political Belief: Tables S7-9

For all three genetic literacy scores, there does not seem to be much information gained by adding religiosity to the model that already includes population, education, and political belief or by adding political belief to the model that already includes population, education, and religiosity. The absolute changes in R^2 are all less than 1%.

Familiarity Score

	Full Model	Political Belief Only	Religiosity Only
**Intercept**	4.507 *** (0.114) p: <0.001	4.407 *** (0.110) p: <0.001	4.630 *** (0.112) p: <0.001
**Population - SPARK**	0.354 *** (0.043) p: <0.001	0.374 *** (0.043) p: <0.001	0.382 *** (0.043) p: <0.001
**Education - High School**	0.715 *** (0.114) p: <0.001	0.703 *** (0.114) p: <0.001	0.715 *** (0.114) p: <0.001
**Education - Undergraduate**	0.960 *** (0.118) p: <0.001	0.933 *** (0.118) p: <0.001	0.977 *** (0.118) p: <0.001
**Education - Graduate**	1.171 *** (0.112) p: <0.001	1.132 *** (0.112) p: <0.001	1.205 *** (0.112) p: <0.001
**Religiosity - Religious, actively practicing**	-0.246 *** (0.056) p: <0.001	--	-0.302 *** (0.054) p: <0.001
**Religiosity - Religious, not actively practicing**	-0.090 (0.053) p: 0.086	--	-0.127 * (0.052) p: 0.015
**Political Belief - Conservative**	0.071 (0.054) p: 0.188	0.031 (0.053) p: 0.561	--
**Political Belief - Liberal**	0.274 *** (0.050) p: <0.001	0.301 *** (0.050) p: <0.001	--
n	3790	3790	3790
R^2	0.088	0.083	0.081
Overall *F* statistic	45.661	57.166	55.212
Overall *F p* value	<0.001	<0.001	<0.001

*** $p < 0.001$; ** $p < 0.01$; * $p < 0.05$.

Table S7: Linear regression model results for the Familiarity GL score by both religiosity and political belief; only political belief; or only religiosity. Entries are the “coefficient (standard error),” and stars indicate statistical significance.

Knowledge Score

	Full Model	Political Belief Only	Religiosity Only
**Intercept**	7.226 *** (0.272) p: <0.001	6.963 *** (0.263) p: <0.001	7.539 *** (0.266) p: <0.001
**Population - SPARK**	2.111 *** (0.102) p: <0.001	2.151 *** (0.102) p: <0.001	2.148 *** (0.102) p: <0.001
**Education - High School**	1.187 *** (0.271) p: <0.001	1.151 *** (0.271) p: <0.001	1.200 *** (0.272) p: <0.001
**Education - Undergraduate**	1.730 *** (0.281) p: <0.001	1.663 *** (0.281) p: <0.001	1.782 *** (0.282) p: <0.001
**Education - Graduate**	2.619 *** (0.267) p: <0.001	2.533 *** (0.267) p: <0.001	2.702 *** (0.268) p: <0.001
**Religiosity - Religious, actively practicing**	-0.534 *** (0.132) p: <0.001	--	-0.592 *** (0.128) p: <0.001
**Religiosity - Religious, not actively practicing**	-0.329 ** (0.125) p: 0.009	--	-0.381 ** (0.124) p: 0.002
**Political Belief - Conservative**	0.409 ** (0.129) p: 0.001	0.329 ** (0.127) p: 0.010	--
**Political Belief - Liberal**	0.615 *** (0.119) p: <0.001	0.680 *** (0.118) p: <0.001	--
n	3790	3790	3790
R^2	0.182	0.178	0.176
Overall *F* statistic	105.002	136.742	134.476
Overall *F* *p* value	<0.001	<0.001	<0.001

*** $p < 0.001$; ** $p < 0.01$; * $p < 0.05$.

Table S8: Linear regression model results for Knowledge GL score by both religiosity and political belief; only political belief; or only religiosity. Entries are the “coefficient (standard error),” and stars indicate statistical significance.

Skills Score

	Full Model	Political Belief Only	Religiosity Only
**Intercept**	1.845 *** (0.143)<br>p: <0.001	1.710 *** (0.138)<br>p: <0.001	1.957 *** (0.140)<br>p: <0.001
**Population - SPARK**	0.859 *** (0.054)<br>p: <0.001	0.884 *** (0.054)<br>p: <0.001	0.889 *** (0.053)<br>p: <0.001
**Education - High School**	0.638 *** (0.142)<br>p: <0.001	0.622 *** (0.143)<br>p: <0.001	0.637 *** (0.143)<br>p: <0.001
**Education - Undergraduate**	0.960 *** (0.148)<br>p: <0.001	0.925 *** (0.148)<br>p: <0.001	0.975 *** (0.148)<br>p: <0.001
**Education - Graduate**	1.175 *** (0.141)<br>p: <0.001	1.126 *** (0.140)<br>p: <0.001	1.208 *** (0.141)<br>p: <0.001
**Religiosity - Religious, actively practicing**	-0.315 *** (0.070)<br>p: <0.001	--	-0.378 *** (0.067)<br>p: <0.001
**Religiosity - Religious, not actively practicing**	-0.135 * (0.066)<br>p: 0.041	--	-0.174 ** (0.065)<br>p: 0.008
**Political Belief - Conservative**	0.034 (0.068)<br>p: 0.611	-0.016 (0.067)<br>p: 0.812	--
**Political Belief - Liberal**	0.259 *** (0.063)<br>p: <0.001	0.295 *** (0.062)<br>p: <0.001	--
n	3790	3790	3790
R^2	0.125	0.120	0.121
Overall *F* statistic	67.595	86.210	86.548
Overall *F p* value	<0.001	<0.001	<0.001

*** $p < 0.001$; ** $p < 0.01$; * $p < 0.05$.

Table S9: Linear regression model results for the Skills GL score by both religiosity and political belief; only political belief; or only religiosity. Entries are the "coefficient (standard error)," and stars indicate statistical significance.

Religious Affiliation and Political Belief

For all three genetic literacy scores, there does not seem to be much information gained by adding religious affiliation to the model that already includes population, education, and political belief or by adding political belief to the model that already includes population, education, and religious affiliation. The absolute changes in R^2 are all less than 1%.
The overall R^2 values are slightly higher for religiosity than for religious affiliation in the models for all three GL scores, but the differences are small.

Familiarity Score

	**Full Model**	**Political Belief Only**	**Religious Affiliation Only**
**Intercept**	4.505 *** (0.114)<br>p: <0.001	4.407 *** (0.110)<br>p: <0.001	4.628 *** (0.112)<br>p: <0.001
**Population - SPARK**	0.364 *** (0.043)<br>p: <0.001	0.374 *** (0.043)<br>p: <0.001	0.397 *** (0.042)<br>p: <0.001
**Education - High School**	0.719 *** (0.114)<br>p: <0.001	0.703 *** (0.114)<br>p: <0.001	0.719 *** (0.114)<br>p: <0.001
**Education - Undergraduate**	0.957 *** (0.118)<br>p: <0.001	0.933 *** (0.118)<br>p: <0.001	0.974 *** (0.118)<br>p: <0.001
**Education - Graduate**	1.159 *** (0.112)<br>p: <0.001	1.132 *** (0.112)<br>p: <0.001	1.192 *** (0.112)<br>p: <0.001
**Religious**	-0.158 *** (0.048)<br>p: <0.001	--	-0.209 *** (0.047)<br>p: <0.001
**Political Belief - Conservative**	0.052 (0.054)<br>p: 0.334	0.031 (0.053)<br>p: 0.561	--
**Political Belief - Liberal**	0.280 *** (0.050)<br>p: <0.001	0.301 *** (0.050)<br>p: <0.001	--
n	3790	3790	3790
R^2	0.086	0.083	0.077
Overall *F* statistic	50.686	57.166	63.579
Overall *F* *p* value	<0.001	<0.001	<0.001
*** $p < 0.001$; ** $p < 0.01$; * $p < 0.05$.			

Table S10: Linear regression model results for the Familiarity GL score by both religious affiliation and political belief; only political belief; or only religious affiliation. Entries are the "coefficient (standard error)," and stars indicate statistical significance.

Knowledge Score

	Full Model	Political Belief Only	Religious Affiliation Only
**Intercept**	7.223 *** (0.272)<br>p: <0.001	6.963 *** (0.263)<br>p: <0.001	7.536 *** (0.266)<br>p: <0.001
**Population - SPARK**	2.124 *** (0.102)<br>p: <0.001	2.151 *** (0.102)<br>p: <0.001	2.166 *** (0.101)<br>p: <0.001
**Education - High School**	1.193 *** (0.271)<br>p: <0.001	1.151 *** (0.271)<br>p: <0.001	1.205 *** (0.272)<br>p: <0.001
**Education - Undergraduate**	1.727 *** (0.281)<br>p: <0.001	1.663 *** (0.281)<br>p: <0.001	1.778 *** (0.282)<br>p: <0.001
**Education - Graduate**	2.603 *** (0.267)<br>p: <0.001	2.533 *** (0.267)<br>p: <0.001	2.686 *** (0.268)<br>p: <0.001
**Religious**	-0.418 *** (0.114)<br>p: <0.001	--	-0.479 *** (0.111)<br>p: <0.001
**Political Belief - Conservative**	0.384 ** (0.128)<br>p: 0.003	0.329 ** (0.127)<br>p: 0.010	--
**Political Belief - Liberal**	0.622 *** (0.119)<br>p: <0.001	0.680 *** (0.118)<br>p: <0.001	--
n	3790	3790	3790
R^2	0.181	0.178	0.175
Overall *F* statistic	119.517	136.742	160.643
Overall *F* *p* value	<0.001	<0.001	<0.001

*** $p < 0.001$; ** $p < 0.01$; * $p < 0.05$.

Table S11: Linear regression model results for the Knowledge GL score by both religious affiliation and political belief; only political belief; or only religious affiliation. Entries are the "coefficient (standard error)," and stars indicate statistical significance.

Skills Score

	Full Model	Political Belief Only	Religious Affiliation Only
**Intercept**	1.843 *** (0.143)<br>p: <0.001	1.710 *** (0.138)<br>p: <0.001	1.954 *** (0.140)<br>p: <0.001
**Population - SPARK**	0.870 *** (0.054)<br>p: <0.001	0.884 *** (0.054)<br>p: <0.001	0.906 *** (0.053)<br>p: <0.001
**Education - High School**	0.643 *** (0.143)<br>p: <0.001	0.622 *** (0.143)<br>p: <0.001	0.642 *** (0.143)<br>p: <0.001
**Education - Undergraduate**	0.957 *** (0.148)<br>p: <0.001	0.925 *** (0.148)<br>p: <0.001	0.971 *** (0.148)<br>p: <0.001
**Education - Graduate**	1.162 *** (0.141)<br>p: <0.001	1.126 *** (0.140)<br>p: <0.001	1.192 *** (0.141)<br>p: <0.001
**Religious**	-0.213 *** (0.060)<br>p: <0.001	--	-0.269 *** (0.058)<br>p: <0.001
**Political Belief - Conservative**	0.012 (0.067)<br>p: 0.857	-0.016 (0.067)<br>p: 0.812	--
**Political Belief - Liberal**	0.266 *** (0.063)<br>p: <0.001	0.295 *** (0.062)<br>p: <0.001	--
n	3790	3790	3790
R^2	0.123	0.120	0.118
Overall *F* statistic	75.925	86.210	101.450
Overall *F p* value	<0.001	<0.001	<0.001
*** $p < 0.001$; ** $p < 0.01$; * $p < 0.05$.			

Table S12: Linear regression model results for the Skills GL score by both religious affiliation and political belief; only political belief; or only religious affiliation. Entries are the "coefficient (standard error)," and stars indicate statistical significance.

Interactions with Education

There are no statistically significant interactions between education and religious affiliation, religiosity, or political belief.

Religious Affiliation

Variable	Familiarity Score	Knowledge Score	Skills Score
**Population**	<0.001	<0.001	<0.001
**Education Level**	<0.001	<0.001	<0.001
**Religious Affiliation**	<0.001	<0.001	<0.001
**Education Level*Religious Affiliation**	0.091	0.709	0.102

Table S13: ANOVA results for interaction test between education level and religious affiliation, with each value reporting statistical significance of the interaction.

	Familiarity Score	Knowledge Score	Skills Score
**Intercept**	4.907 *** (0.165)<br>p: <0.001	7.730 *** (0.393)<br>p: <0.001	2.306 *** (0.204)<br>p: <0.001
**Population - SPARK**	0.320 *** (0.041)<br>p: <0.001	1.949 *** (0.099)<br>p: <0.001	0.837 *** (0.051)<br>p: <0.001
**Education - High School**	0.347 * (0.176)<br>p: 0.050	0.848 * (0.421)<br>p: 0.044	0.216 (0.218)<br>p: 0.324
**Education - Undergraduate**	0.710 *** (0.188)<br>p: <0.001	1.518 *** (0.447)<br>p: <0.001	0.673 ** (0.232)<br>p: 0.004
**Education - Graduate**	0.950 *** (0.175)<br>p: <0.001	2.616 *** (0.417)<br>p: <0.001	0.772 *** (0.216)<br>p: <0.001
**Religious**	-0.637 ** (0.213)<br>p: 0.003	-0.746 (0.507)<br>p: 0.141	-0.834 ** (0.263)<br>p: 0.002
**Education - High School*Religious**	0.541 * (0.226)<br>p: 0.017	0.429 (0.540)<br>p: 0.427	0.635 * (0.280)<br>p: 0.024
**Education - Undergraduate*Religious**	0.388 (0.237)<br>p: 0.102	0.302 (0.566)<br>p: 0.594	0.521 (0.294)<br>p: 0.076
**Education - Graduate*Religious**	0.399 (0.223)<br>p: 0.074	0.172 (0.533)<br>p: 0.747	0.661 * (0.277)<br>p: 0.017
n	4057	4057	4057
R^2	0.070	0.156	0.108
Overall *F* statistic	38.068	93.237	60.968
Overall *F* *p* value	<0.001	<0.001	<0.001
*** $p < 0.001$; ** $p < 0.01$; * $p < 0.05$.			

Table S14: Linear regression model results for each GL score by population, education, and interaction between education and religious affiliation. Entries are the “coefficient (standard error),” and stars indicate statistical significance.

Religiosity

Variable	Familiarity Score	Knowledge Score	Skills Score
Population	<0.001	<0.001	<0.001
Education Level	<0.001	<0.001	<0.001
Religiosity	<0.001	<0.001	<0.001
Education Level*Religiosity	0.338	0.950	0.202

Table S15: ANOVA results for interaction test between education level and religiosity, with each value reporting statistical significance of the interaction.

	Familiarity Score	Knowledge Score	Skills Score
**Intercept**	4.911 *** (0.164) p: <0.001	7.736 *** (0.393) p: <0.001	2.311 *** (0.204) p: <0.001
**Population - SPARK**	0.307 *** (0.042) p: <0.001	1.933 *** (0.099) p: <0.001	0.819 *** (0.051) p: <0.001
**Education - High School**	0.349 * (0.176) p: 0.048	0.850 * (0.421) p: 0.043	0.219 (0.218) p: 0.316
**Education - Undergraduate**	0.714 *** (0.187) p: <0.001	1.523 *** (0.447) p: <0.001	0.680 ** (0.232) p: 0.003
**Education - Graduate**	0.954 *** (0.175) p: <0.001	2.621 *** (0.417) p: <0.001	0.778 *** (0.216) p: <0.001
**Religiosity - Religious, not actively practicing**	-0.555 * (0.248) p: 0.025	-0.837 (0.592) p: 0.157	-0.909 ** (0.307) p: 0.003
**Religiosity - Religious, actively practicing**	-0.727 ** (0.256) p: 0.004	-0.645 (0.610) p: 0.291	-0.750 * (0.316) p: 0.018
**Education - High School*Religiosity - Religious, not actively practicing**	0.517 * (0.263) p: 0.049	0.618 (0.627) p: 0.324	0.780 * (0.325) p: 0.016
**Education - Undergraduate*Religiosity - Religious, actively practicing**	0.337 (0.283) p: 0.233	0.057 (0.675) p: 0.933	0.273 (0.350) p: 0.436
**Education - Graduate*Religiosity - Religious, not actively practicing**	0.408 (0.260) p: 0.116	0.385 (0.620) p: 0.535	0.877 ** (0.322) p: 0.006
n	4057	4057	4057
R^2	0.073	0.156	0.111
Overall *F* statistic	26.620	62.504	42.149
Overall *F p* value	<0.001	<0.001	<0.001

*** $p < 0.001$; ** $p < 0.01$; * $p < 0.05$.

Table S16: Linear regression model results for each GL score by population, education, and interaction between education and religiosity. Entries are the “coefficient (standard error),” and stars indicate statistical significance.

Political Belief

Variable	Familiarity Score	Knowledge Score	Skills Score
Population	<0.001	<0.001	<0.001
Education Level	<0.001	<0.001	<0.001
Political Belief	<0.001	<0.001	<0.001
Education Level*Political Belief	0.578	0.743	0.372

Table S17: ANOVA results for interaction test between education level and political belief, with each value reporting statistical significance of the interaction.

	Familiarity Score	Knowledge Score	Skills Score
**Intercept**	4.594 *** (0.161)<br>p: <0.001	7.087 *** (0.383)<br>p: <0.001	1.685 *** (0.201)<br>p: <0.001
**Population - SPARK**	0.373 *** (0.043)<br>p: <0.001	2.152 *** (0.102)<br>p: <0.001	0.884 *** (0.054)<br>p: <0.001
**Education - High School**	0.496 ** (0.171)<br>p: 0.004	1.035 * (0.407)<br>p: 0.011	0.653 ** (0.214)<br>p: 0.002
**Education - Undergraduate**	0.817 *** (0.180)<br>p: <0.001	1.704 *** (0.430)<br>p: <0.001	0.781 *** (0.226)<br>p: <0.001
**Education - Graduate**	0.910 *** (0.170)<br>p: <0.001	2.310 *** (0.405)<br>p: <0.001	1.225 *** (0.213)<br>p: <0.001
**Political Belief - Conservative**	-0.275 (0.272)<br>p: 0.312	0.517 (0.648)<br>p: 0.425	-0.028 (0.341)<br>p: 0.935
**Political Belief - Liberal**	-0.061 (0.249)<br>p: 0.808	0.141 (0.594)<br>p: 0.812	0.383 (0.313)<br>p: 0.220
**Education - High School*Political Belief - Conservative**	0.325 (0.287)<br>p: 0.258	-0.271 (0.684)<br>p: 0.692	-0.014 (0.360)<br>p: 0.968
**Education - Undergraduate*Political Belief - Liberal**	0.271 (0.274)<br>p: 0.322	0.269 (0.654)<br>p: 0.681	0.206 (0.344)<br>p: 0.549
**Education - Graduate*Political Belief - Conservative**	0.376 (0.284)<br>p: 0.186	-0.036 (0.676)<br>p: 0.958	-0.063 (0.356)<br>p: 0.860
n	3790	3790	3790
R^2	0.084	0.179	0.122
Overall *F* statistic	28.968	68.618	43.650
Overall *F p* value	<0.001	<0.001	<0.001
*** $p < 0.001$; ** $p < 0.01$; * $p < 0.05$.			

Table S18: Linear regression model results for each GL score by population, education, and interaction between education and political belief. Entries are the “coefficient (standard error),” and stars indicate statistical significance.
