# Supplementary material for: Interaction of identity and beliefs with genetic literacy
Source: Am J Hum Genet. 2025 Dec 22;113(1):16–28. doi: 10.1016/j.ajhg.2025.11.014 (PMC12817316; doi:10.1016/j.ajhg.2025.11.014)
Supplement: Document S2. Article plus supplemental information [file mmc2.pdf]

ARTICLE

Interaction of identity and beliefs with genetic literacy

Authors

Gabriela M. Ramírez Renta, India D. Little, Laura M. Koehly, ..., Jessica Butts, Jordan Lundeen, Chris Gunter

Correspondence

chris.gunter@nih.gov

To understand how people relate to genetics, we must measure more than knowledge. Here, we show from over 4,000 participants that multiple personal beliefs have significant effects on genetic literacy, particularly confidence in one's own genetic knowledge. This suggests that improving confidence is crucial to uptake of genetics and genomics.

Ramírez Renta et al., 2026, The American Journal of Human Genetics *113*, 16–28
January 8, 2026 Published by Elsevier Inc. on behalf of American Society of Human Genetics.
https://doi.org/10.1016/j.ajhg.2025.11.014

CellPress

ARTICLE

Interaction of identity and beliefs with genetic literacy

Gabriela M. Ramírez Renta,[1,5] India D. Little,[2,6] Laura M. Koehly,[1] Anna J. Hilliard,[1,7] Kaylee L. Foor,[1,8] Jessica Butts,[3] Jordan Lundeen,[3] and Chris Gunter[1,4,*]

Summary

Genetic literacy goes beyond knowledge of genetic terms, as it requires sufficient skills and understanding to effectively facilitate health-related decision-making and participation in social discussions about genetic issues. Personal identity and beliefs have been shown to affect how individuals interact with new information, but rarely in the context of genetic literacy. In 2021, we created and disseminated a survey to two separate samples: 2,050 members of the US general public and 2,023 participants in a large genetic research study. We assessed genetic literacy through three components: subjective knowledge (Familiarity), objective knowledge (Knowledge), and knowledge comprehension (Skills), making this one of the only large-scale surveys to assess comprehension as a part of genetic literacy. We hypothesized that additional measures of identity and belief factors would enable a better understanding of how individuals process and retain genetic information. We found that confidence in one's genetic knowledge was the strongest predictor of positive scores in all three components, controlling nearly 25% of the variance in scores, while perceived importance of genetic information had a positive but weaker relationship to scores. This suggests that improving confidence, not just providing knowledge, is an important part of increasing uptake of genetics in various applications. Further, we found that multiple self-described beliefs had mixed predictive effects on all three of our genetic literacy subscales. These findings demonstrate the complexity inherent in endeavors to raise genetic literacy in the US population as an example, as well as the importance of context-specific genetics communication.

Introduction

As genetics and genomics is more readily applied to health and society, such as increased use of genetic testing in clinical and direct-to-consumer contexts, the need to understand and apply these concepts continues to grow. A 2023 study documented over 37,000 current unique genetic tests in the US as entered into the National Institutes of Health (NIH) Genetic Testing Registry,[1] with an average of 5,000 entries every year. This flood of information necessitates an ability for both patients/consumers and providers to comprehend genetics- and genomics-related concepts. The construct of genetic literacy (GL) is not simply objective genetic knowledge but an assessment of an individual's understanding of basic genetics concepts as well as their ability to apply them to their own health.[2] This construct was designed to measure how prepared a population is to receive and interpret genetic and genomic information.[3] To capture GL, measures must include a knowledge comprehension component, as this capacity is included in our definition of GL.[3] Unfortunately, many measures widely in use[4–6] do not include this component.

Recognizing there is a growing need to evaluate the capacity of individuals to acquire and process genetic information, we previously adapted a survey measure developed by Abrams et al.[7] with three components: subjective knowledge (called Familiarity), objective knowledge (called Knowledge), and knowledge comprehension (called Skills). We then repeated the administration of a GL survey measure from 2013 to two groups in 2021: a general population (GenPop) sample and a genetics research participant sample through the Simons Powering Autism Research for Knowledge (SPARK) study.[8] We found that the GL of the GenPop rose slightly from 2013 to 2021, and the research participants in SPARK scored higher than both GenPop groups,[8] suggesting that those motivated to participate in a genetics research study had higher literacy and/or they learned through participating in the study. However, the average scores do not capture psychosocial factors that might be influencing GL as a construct, motivating us to continue exploring with survey data.

Multiple studies have demonstrated that individuals process new information or ideas according to pre-existing beliefs,[9,10] consistent with the cultural cognition theory, which states that individuals' cultural values shape their interpretations of information and situations.[11] This differs from (but can appear similar to) confirmation bias, which is processing novel information according to

[1]Social and Behavioral Research Branch, National Human Genome Research Institute, Bethesda, MD 20892, USA; [2]Genetic Counseling Training Program, National Human Genome Research Institute, Johns Hopkins Bloomberg School of Public Health, Baltimore, MD 21287, USA; [3]The Emmes Company, Rockville, MD 20850, USA; [4]Office of the Director, National Human Genome Research Institute, Bethesda, MD 20892, USA
[5]Present address: University of Colorado, Aurora, CO 80045, USA
[6]Present address: Atrium Health, Levine Children's Specialty Center, Medical Genetics, Charlotte, NC 28203, USA
[7]Present address: University of Pittsburgh, Pittsburgh, PA 15261, USA
[8]Present address: Carnegie Mellon University, Pittsburgh, PA 15213, USA
*Correspondence: chris.gunter@nih.gov
https://doi.org/10.1016/j.ajhg.2025.11.014.
Published by Elsevier Inc. on behalf of American Society of Human Genetics.
This is an open access article under the CC BY license (http://creativecommons.org/licenses/by/4.0/).

what someone already believes as opposed to fitting assessments of evidence to someone's cultural predispositions.[9] With that said, there is a complex relationship between science knowledge, confidence, and identity factors. One recurring theme is that both overconfidence in one's own knowledge and reporting a less positive attitude toward science peak in individuals with intermediate levels of true scientific knowledge rather than in either low- or high-knowledge groups.[12] Previous work with our measure has shown that there is an indirect relationship between familiarity (what one thinks one knows) and objective knowledge; this relationship is partially mediated by skills (knowledge comprehension).[7] This implies that measurement of skills needed to synthesize and apply genetic information may be an important tool to ensure that high familiarity is not an expression of overconfidence in one's objective knowledge. Genetic knowledge may not increase even after genetic testing[13] or clinical services,[14,15] and groups that elect to decline or receive genetics services may not have significant differences in genetic knowledge.[15] Again, this led us to examine the possibility that some identifiable cultural factors or beliefs could contribute to processing and application of genetic information as captured by our GL measure.

In addition to education level, we therefore chose to examine two continuous, subjective measures: perceptions of the importance of genetic knowledge and confidence in one's own genetic knowledge. We also measured four categorical variables of identity and beliefs: self-described political belief, self-described religious affiliation, religiosity, and conflict between religion and science.

Self-described political belief has often been assessed in the context of trust in science or scientists, with a recent large study showing that geneticists are among the scientific professions with the least differential in trust between "liberals" and "conservatives" in the US.[16] A 2021 US-based survey concluded "scientists and science communicators must consider political polarization when engaging the public," based on both similarity and divergence of priorities based on "political leaning" of respondents (Heslop et al., https://mediaengagement.org/research/communicating-science-across-political-divides/). As political belief represents another factor influencing the acceptance of new scientific information and attitudes toward personal genomic testing,[17] for example, we assessed this variable in our study populations.

Depending on how the question is asked and which measures are used, the reported relationship between religious beliefs and genetics has ranged from indirect but negative[18] to unclear and mixed[19]; however, there is agreement that these beliefs can act as a moderator for knowledge and attitudes.[19] Parrott et al.[20] reported from their US-based survey (n = 858) that religiosity was only slightly negatively correlated with objective genetic knowledge and that religiosity does not predetermine pessimistic attitudes about genes and health. In a larger, multi-country survey (n = 5,404), Chapman et al.[5] reported a significantly lower score in genetic knowledge between self-described believers and non-believers in a primarily Russian-language population; however, neither of these two studies tested knowledge comprehension. In 2018, the Wellcome Global Monitor 2018 (https://cms.wellcome.org/sites/default/files/wellcome-global-monitor-2018.pdf) surveyed over 140,000 people worldwide and found that 76% of North Americans said they identify with a specific religion. Of those, 57% reported that science has disagreed with the teaching of their religion. The authors point out that "notably, Northern America is the only high-income region in which people who say they have a religion are substantially more likely to say they believe their religion's teaching over science, in cases of disagreement." Relevant to our study of a US population, the 57% finding was primarily driven by US participants, where 60% reported they would believe religious teachings and 32% said they would believe science.

Here, we report the interactions between measures for multiple identity and belief factors and our three primary GL subscales in a 2021 survey of over 4,000 US respondents. Evaluating the relative contributions of these beliefs can be helpful in tailoring communications about genetics and "meeting people where they are"[21] rather than assuming a simple deficit of knowledge that must be provided by professionals.[22]

Subjects and methods

The Genetic and Autism Literacy Survey (GALS, described below) was conducted entirely online and was designed to have a completion time of approximately 25 min. The research protocol was deemed exempt by the Office of Research Protection of the NIH and approved by the Institutional Review Board at the NIH (protocol 000399).

Language preferences

We realize that the phrases used to characterize autism are fluid and personal and that they should be based on what participants in the research prefer to express themselves. We used the terms "autism," "autism spectrum disorder," or "ASD" to denote the condition in 2021 and "autistic" to refer to individuals participating in this study. However, we acknowledge that language is continuously evolving and that since the time the survey was conducted, community-favored guidelines for discussing autism-related information have diverged from some of the terms used.[23]

Participants and survey development

2021 GenPop sample

As described in Little et al.,[8] in April 2021 the GALS was distributed to a sample of 2,050 people recruited from a respondent panel generated by a third-party contractor. To match the sample distribution in Abrams et al.,[7] the sample over-represents Black participants (27.5%).

SPARK sample

From September through November 2021, GALS was distributed to a sample of 2,023 autistic individuals and families with autism

participating in the SPARK Research Match program.[8] To resemble previous sample distributions, the sample again over-represents Black participants (20.5%). Because 16 SPARK samples did not have educational-level data, we removed them from these analyses.

Survey measures

Genetic and autism literacy scale

GALS consists of three subscales[8]: (1) Familiarity, which asks participants to rate their familiarity with eight genetics-related terms on a scale of 1 (not at all familiar) to 7 (completely familiar), and the overall score is presented as an average, creating a range of 1–7; (2) Knowledge, where participants are presented with 16 technical genetic statements and asked to respond with "true," "false," or "don't know" for each statement, creating a final score range of 0–16; and (3) Skills, where participants read a one-page infographic describing genetic influences on autism and are asked six questions directly related to the information presented, creating a range of 0–6. As reported in the previous work, the Familiarity scale has a Cronbach's alpha of 0.923; and the Knowledge and Skills scales have Kuder-Richardson 20 scales (appropriate for dichotomously scored items) of 0.725 and 0.654, respectively.

Perceived importance of genetic knowledge

This question from Kaphingst et al.[24] asked "how important is it to you that you learn more about how your genes—the characteristics that are passed down from generation to generation—affect your risk of developing certain health conditions?" To express how significant this element was to them, participants had to use a Likert scale with seven choices ranging from "very unimportant" to "very important."

Confidence in genetic knowledge

We used the six questions on "subjective knowledge in genetics" from Adams et al.[14] to ask the participants how confident they were about their knowledge of genetics and their ability to seek information if needed. The six statements include "you are confident in your ability to understand information about genetics" and "it would be easy for you to get information about genetics if you wanted to." Participants chose a value from a Likert scale with five choices ranging from "strongly disagree" to "strongly agree." In this dataset, the scale demonstrated good internal consistency with a Cronbach's alpha of 0.87.

Religious beliefs, religiosity, and science

As we were interested in the relationship between GL and not just religion but a potential interaction between religion and science, we adapted two questions from the Wellcome Global Monitor 2018 (https://cms.wellcome.org/sites/default/files/wellcome-global-monitor-2018.pdf), and added a third on religiosity.

1. Self-described religious group. Participants were asked to choose the religious group which most adequately described them from a list of 13 options that included "Baptist," "Buddhist," "Catholic," "Eastern Orthodox," "Hindu," "Jewish," "Mormon," "Muslim," "Pentecostal," "Protestant-Memorial, Lutheran, Presbyterian, Episcopal," "other Christian," "other non-Christian," and "none." For statistical analyses, participants that selected "none" were considered "non-religious" and all other options were considered "religious."
2. Religiosity. Participants were asked "how often do you go to religious services?" Response options were a scale of 1–7 based on timing. For statistical analyses, we combined this answer with the religious affiliation above, using final categorizations of "Non-religious," "Religious, not actively practicing," and "Religious, actively practicing." If they responded they were "non-religious," they were categorized as "Non-religious" again here. Those who selected "religious" and the timing options "several times a year," "about once or twice a year," or "never" were categorized as "Religious, not actively practicing." Those who selected "religious" and one of "about once a month," "2–3 times a month," "once a week," or "several times a week" were categorized as "Religious, actively practicing."
3. Conflict between religion and science. Participants were asked two questions on decisions they made in accordance with their beliefs. One of them inquired if their religion's teachings had ever disagreed with science, with options of "yes," "no," "don't know," and "prefer not to say." If they answered "yes," they were asked "generally speaking, when science disagrees with the teachings of your religion, what do you believe? Science or the teachings of your religion?" Response alternatives were "science," "the teachings of your religion," "it depends," "don't know," and "prefer not to say." We excluded "it depends," "don't know," and "prefer not to say" responses from our analyses.

Self-described political belief

We asked participants to choose their self-identified political belief from seven options: 1 (extremely conservative), 2 (conservative), 3 (slightly conservative), 4 (moderate, middle of the road), 5 (slightly liberal), 6 (liberal), or 7 (extremely liberal). We clustered the beliefs into three groups for statistical analyses: "conservative" containing 1–3; "moderate" or 4; and "liberal" containing 5–7.

Statistical analysis

We completed analyses using R (v.4.2.2, https://www.r-project.org/) and SAS (version 9.4) statistical software. All continuous measures are summarized using the following descriptive statistics: *n* (non-missing sample size), mean, standard deviation, median, minimum, and maximum. We report the frequency and percentage (based on population sample size) of observed levels for all categorical measures. We used linear regression models to explore the relationship between GL subscale scores and the identity/belief measures. Population (GenPop vs. SPARK) and education have previously been shown as statistically significant contributors to GL in Little et al.,[8] with SPARK having higher scores and education levels correlated with higher scores; therefore, population and education are included in all models. For any given model, we exclude participants with missing data (i.e., GL scores, education level, or identity/belief measure). Given that these analyses are exploratory, we did not make adjustments for multiple comparisons.[25] To examine differences in GL scores across political belief groups (conservative, moderate, and liberal), we fitted linear regression models that included population and education as covariates. We then conducted post hoc pairwise comparisons of the political belief coefficients using Tukey-adjusted multiple comparisons as implemented in the *multcomp* package in R (supplemental information). To evaluate whether the effect of education on GL varied by political or religious factors, we extended the regression models to include

Table 1. Summary of continuous measures by population

Variable	Statistic	GenPop ($n = 2{,}050$)	SPARK ($n= 2{,}007$)
Perceived importance of genetic knowledge (one question, Likert scale 1–7)	*n*	2,050	2,007
	mean	5.5	6.1
	standard deviation	1.6	1.5
	median	6.0	7.0
	minimum	1.0	1.0
	maximum	7.0	7.0
Confidence in genetic knowledge (average of six questions, Likert scale 1–5)	*n*	2,050	2,007
	mean	3.6	3.9
	standard deviation	0.8	0.8
	median	3.7	4.0
	minimum	1.0	1.0
	maximum	5.0	5.0

interaction terms (education × political belief, education × religiosity, and education × religious affiliation). Interaction effects were tested using model-based *F* tests within the linear regression framework (supplemental information).

The fact that we see highly significant results for most of our unstandardized regressions is likely primarily due to the large sample sizes of our study.[26] Code debugging and visualization refinement were performed with assistance from Claude AI (https://www.anthropic.com/claude/opus), which provided guidance on syntax errors and plot formatting. All statistical decisions and interpretations were made by the authors.

Results

Descriptive statistics for each identity/belief measure, along with GL scores, are presented in Tables 1 and 2. Table 1 provides detailed descriptive statistics for continuous measures across various populations. Table 2 offers a summary of categorical measures by population, including the distribution of responses for these measures. A majority of the participants in both groups described themselves as part of a religious group (76% of GenPop and 66% of SPARK, respectively). Self-described belief was closer to evenly distributed among three options offered to the GenPop group, while 40.2% of the SPARK participants described themselves as liberal and only 17.6% conservative. As reported in Little et al.,[8] we also see a difference in education level between the two populations, shown in Tables 3, 4, and 5 as “model 1.” Education levels are compared to having less than high school as the reference. Tables 3, 4, and 5 further show the regression results for each of the identity/belief scales against the three areas of our GALS measure: familiarity/subjective knowledge in Table 3; objective knowledge in Table 4; and skills/knowledge comprehension in Table 5. Each table presents five linear regression models predicting GL Familiarity scores, with each cell showing the unstandardized regression coefficient and its standard error in parentheses. In Table 3 as an example, these coefficients represent the expected change in Familiarity scores (measured on a 1–7 scale) for a one-unit increase in continuous predictors, or the difference from the reference category for categorical variables; for example, the coefficient of 0.343 for population: SPARK in model 1 indicates that SPARK participants score 0.343 points higher on average than the GenPop reference group with a standard error of 0.041 ($p < 0.001$). The R^2 values show that model 3, which includes confidence in genetic knowledge, explains 25.5% of the variance in Familiarity scores, representing a substantial improvement ($\Delta R^2 = 0.191$) over the base model’s 6.4% explained variance. We now discuss each of the additional identity and belief measures separately.

Effects of perceived importance of and confidence in genetic knowledge

Figure 1 shows the score for perceived importance of genetic knowledge, a one-question Likert scale with seven options, by each GL score (Familiarity, Knowledge, and Skills) for each population (GenPop and SPARK). Table S1 presents the regression results for each GL score by population, education, and perceived gene importance score. Perceived importance was a statistically significant positive predictor for all three GL subscales after adjusting for population and education. A one-unit increase of this measure resulted in an increase in all the GL subscales scores (Familiarity [0.196], Knowledge [0.265] and Skills [0.085], seen as model 2 in Tables 3, 4, and 5), indicating that when individuals say they place increased importance in learning about genetic topics, they have a significantly higher GL level in all three domains.

Similar figures for the remaining measures and detailed regression tables can be found in the supplemental information. Figure S1 and Table S2 contain data for the

Table 2. **Summary of categorical measures by population**

Measure	Response	GenPop (n = 2,050)		SPARK (n = 2,007)	
		n	%[a]	n	%[a]
Self-described religious affiliation	religious	1,559	76.0	1,324	66.0
	non-religious	491	24.0	683	34.0
Religiosity	religious, actively practicing	821	40.0	580	28.9
	religious, not actively practicing	738	36.0	744	37.1
	non-religious	491	24.0	683	34.0
Conflict between religion and science	religion	232	11.3	113	5.6
	science	203	9.9	309	15.4
Self-described political belief	liberal	611	29.8	814	40.6
	moderate	748	36.5	572	28.5
	conservative	691	33.7	354	17.6
Education level	early schooling	106	5.2	50	2.5
	high school	697	34.0	575	28.6
	undergraduate	350	17.1	423	21.1
	graduate	897	43.8	959	47.8

[a]Denominators for percentages are the total sample size for the given population; therefore, percentages will not add up to 100 given missing data for some measures.

confidence in genetic knowledge score (an average of six questions on a Likert scale with five options) relative to each GL score across different populations and regression results for each GL score. As seen in model 3 in Tables 3, 4, and 5, after adjusting for population and education, confidence in genetic knowledge emerged as a statistically significant positive predictor for all three GL subscales. Specifically, a one-unit increase in confidence in genetic knowledge corresponded to increases in all of the GL subscale scores of Familiarity (0.753), Knowledge (1.396), and Skills (0.299). This suggests that greater interest in learning and enhanced confidence in genetic knowledge contribute to higher levels of GL. In fact, this confidence variable accounted for more of the variance in scores than any other measure we tested, being approximately 25% of the variance in the Familiarity and Knowledge scores after correction for population and education (R^2 for model 3 in Tables 3, 4, and 5).

Religious identity/beliefs and genetic literacy scale

Self-described religious affiliation

We asked participants in both our research samples whether they self-identify as having a preferred religious group (Table 2), with 76% of the GenPop and 66% of the SPARK sample indicating they did. Figure S2 shows GL scores by religious affiliation (religious vs. non-religious) in both populations. Table S3 presents the regression results for each subscale by population, education, and religious affiliation. Declaring that one has a religious affiliation was a statistically significant negative predictor for all three GL subscales after adjusting for population and education (model 4 in Tables 3, 4, and 5), with religious participants having lower scores for Familiarity (−0.21), Knowledge (−0.472), and Skills (−0.238) subscales compared to participants who described themselves as non-religious.

Religiosity

Given that previous work in this area focused on religiosity as the variable interacting with GL, rather than religious affiliation, we also asked our participants about their frequency of attending religious services. Figure S3 shows GL scores by religiosity (non-religious vs. religious; not actively practicing vs. religious, actively practicing) in both populations. Table S4 presents the regression results for each subscale by population, education, and religiosity group. As seen there and in model 5 of Tables 3, 4, and 5, when compared to the scores of the non-religious group, participants in both the religious practice categories had significantly lower scores for all three GL subscales after adjusting for population and education. Participants in the religious, actively practicing group had a larger decrease compared to the non-religious group in all three GL scores than the religious, not actively practicing group.

Conflict between religion and science

We asked participants in both of our research samples who identified as having a religious group whether they believed their religion or science when there was a disagreement between the two. In the first question, "has science has ever disagreed with the teachings of your religion?," 34.4% (705/2,050) of the GenPop and 32.7% (656/2,007) of the SPARK samples answered yes. For our second question ("generally speaking, when

Table 3. Linear regression models predicting familiarity/subjective knowledge scores (range: 1–7) with identity and belief variables

Familiarity/subjective knowledge (range: 1–7)	Model 1	Model 2	Model 3	Model 4	Model 5
Constant	4.519*** (0.105)	3.508*** (0.122)	2.094*** (0.12)	4.652*** (0.109)	4.656*** (0.109)
Population: SPARK (reference: GenPop)	0.343*** (0.041)	0.234** (0.041)	0.120*** (0.037)	0.320*** (0.041)	0.308*** (0.041)
Education (reference: less than high school)					
High school	0.658*** (0.111)	0.615*** (0.108)	0.412*** (0.099)	0.679*** (0.111)	0.674*** (0.111)
Undergraduate	0.903*** (0.115)	0.833*** (0.112)	0.612*** (0.103)	0.936*** (0.115)	0.937*** (0.115)
Graduate	1.151*** (0.109)	1.069*** (0.106)	0.749*** (0.098)	1.184*** (0.109)	1.195*** (0.109)
Perceived importance of genetic knowledge	–	0.196*** (0.013)	–	–	–
Confidence in genetic knowledge	–	–	0.753*** (0.023)	–	–
Religious affiliation (reference: none)	–	–	–	−0.21*** (0.046)	–
Religious activity (reference: no religious affiliation)					
Religious, not actively practicing	–	–	–	–	−0.125* (0.051)
Religious, actively practicing	–	–	–	–	−0.306*** (0.052)
R^2	0.064	0.114	0.255	0.068	0.072
ΔR^2 from model 1	–	0.050	0.191	0.004	0.008
No. of observations	4,057	4,057	4,057	4,057	4,057

Each cell contains the unstandardized regression coefficient (standard error in parentheses), representing the change in Familiarity score associated with a one-unit increase in continuous predictors or the difference from the reference category for categorical predictors. R^2 indicates the proportion of variance in Familiarity scores explained by each model. ΔR^2 from model 1 shows the additional variance explained by adding predictors beyond the base model. $n = 4{,}057$. ***$p < 0.001$; **$p < 0.01$; *$p < 0.05$.

science disagrees with the teachings of your religion, what do you believe? Science or the teachings of your religion?"), the values are shown in Table 2. Of those who indicated making a choice, 53.3% of the GenPop (232/435) and 26.8% of the SPARK participants (113/422) said they chose to believe religion when there is a disagreement. Figure S4 shows GL scores by conflict between religion and science (Religion or Science) in both populations. Table 6 presents the regression results and shows that choosing "Science" was a statistically significant positive predictor for each GL score after adjusting for population and education, resulting in higher GL scores for Familiarity (0.332), Knowledge (1.018), and Skills (0.401) compared to participants that chose to follow the teachings of their religion. In contrast to our other modifier tests, the regression did not show a significant association with education in all of the tested groups, likely due to the smaller sample size ($n = 857$ in total).

Political beliefs and genetic literacy scale

We asked participants in each of our research samples about their self-described political belief, with options being conservative, liberal, and moderate (Table 2). Figure S5 depicts the distribution of GL subscales and participants' self-described political belief in both populations. Table S5 presents the regression results for each GL score by population, education, and political belief; we regressed the two ends of the ideological spectrum against the "moderate" values. Declaring a "conservative" political belief was only a statistically significant positive predictor for the Knowledge score after adjusting for population and education, while a "liberal" self-described belief was a statistically significant positive predictor for all three GL subscales when compared to participants self-identifying as "moderate." Additionally, we conducted pairwise comparisons between the levels of political belief adjusting for multiple comparisons using Tukey's method (Table S6). The results were similar but allowed us to add the direct comparison between the two ends of the spectrum, showing that participants with a "liberal" political belief have highly statistically significant ($p < 0.001$) higher GL scores for Familiarity and Skills compared to participants with a "conservative" political belief but only a statistically significant ($p < 0.05$) difference in the Knowledge score.

Overall and interacting effects of identity and belief models

Finally, we examined the effect of multiple regression for the continuous variables related to all three GL subscales. Because religious affiliation and religiosity have overlapping definitions, we did not include them in the same model. We saw little value in adding religiosity to a model containing political belief, or vice versa (both models already contained population and education, Tables S7–S12). We also tested for but found no statistically significant interactions between education and any of religious

Table 4. Linear regression models predicting knowledge/objective knowledge scores (range: 0–17) with identity and belief variables

Knowledge/objective knowledge (range: 0–16)	Model 1	Model 2	Model 3	Model 4	Model 5
Constant	7.268*** (0.251)	5.901*** (0.297)	2.770*** (0.301)	1.951*** (0.135)	1.956*** (0.135)
Population: SPARK (reference: GenPop)	2.003*** (0.098)	1.856** (0.099)	1.589*** (0.094)	0.834*** (0.051)	0.819*** (0.051)
Education (reference: less than high school)					
High school	1.070*** (0.264)	1.012*** (0.262)	0.613*** (0.248)	0.598*** (0.137)	0.592*** (0.137)
Undergraduate	1.627*** (0.274)	1.532*** (0.272)	1.087*** (0.258)	0.975*** (0.142)	0.976*** (0.142)
Graduate	2.628*** (0.260)	2.517*** (0.258)	1.882*** (0.246)	1.175*** (0.135)	1.179*** (0.135)
Perceived importance of genetic knowledge	–	0.265*** (0.031)	–	–	–
Confidence in genetic knowledge	–	–	1.396*** (0.058)	–	–
Religious affiliation (reference: none)	–	–	–	−0.472*** (0.109)	–
Religious activity (reference: no religious affiliation)					
Religious, not actively practicing	–	–	–	–	−0.363** (0.122)
Religious, actively practicing	–	–	–	–	−0.593*** (0.125)
R^2	0.151	0.166	0.256	0.155	0.156
ΔR^2 from model 1	–	0.015	0.105	0.004	0.005
No. of observations	4,057	4,057	4,057	4,057	4,057

Each cell contains the unstandardized regression coefficient (standard error in parentheses), representing the change in Knowledge score associated with a one-unit increase in continuous predictors or the difference from the reference category for categorical predictors. R^2 indicates the proportion of variance in Knowledge scores explained by each model. ΔR^2 from model 1 shows the additional variance explained by adding predictors beyond the base model. $n = 4{,}057$. ***$p < 0.001$; **$p < 0.01$; *$p < 0.05$.

affiliation, religiosity, or political belief (Tables S13–S18), again on all three GL subscales.

To visualize relative effects of each of the identity and belief measures we tested, we plotted all of the test results against the three subscales of our measure in Figure 2, with asterisks present or absent for level of statistical significance. Unstandardized coefficients, reported in our regression tables, preserve the original measurement scales and are essential for understanding the practical magnitude of effects—for instance, how a one-unit change in education level translates to changes in GL scores. However, standardized coefficients in our forest plots facilitate direct comparison of relative importance across predictors measured on different scales (e.g., population, education, and genetic knowledge confidence), allowing us to identify which factors are the strongest drivers of GL outcomes in our large sample. To include political ideology, we had to include the "prefer not to answer" category; here, we also could not include belief in science because of the much lower sample size for that measure.

After standardization, the relative differences in effects of education levels and identity/belief measures on each of the GL subscales are more clear: for example, scores for those with a high school education were highly significantly different from no schooling for Familiarity, but not for Knowledge, and only just significantly different in Skills. As one might predict, genetic knowledge confidence demonstrates a stronger correlation with subjective knowledge than either objective knowledge or skills. Finally, those who preferred not to answer their political ideology had a consistently more negative relationship with all three GL measures than the "moderate" group to which they were compared.

Discussion

Our study demonstrated that education levels, perceived importance of genetic knowledge, confidence in one's own genetic knowledge, some political beliefs, and a belief in science over religion when the two were perceived to be in conflict are all significant positive predictors for GL scores.

As opposed to only measuring genetic knowledge, we continue to use the GALS to capture multiple dimensions of the construct of GL. An important part of understanding this construct is to characterize the effects of multiple personal identity or belief components on facets of GL. Previous work has also shown that levels of general education, science education, and science literacy do not show the same levels of correlation or polarization with either political or religious identity and therefore should not be substituted for one another.[27] In addition, for GL, as with many related constructs like health or science literacy, processing of scientific information by those who have not trained as scientists involves various and sometimes conflicting cognitive mechanisms.[11] Our demonstration that individual identity and belief constructs

Table 5. Linear regression models predicting skills/knowledge comprehension scores (range: 0–6) with identity and belief variables

Skills/knowledge comprehension (range: 0–6)					
	Model 1	Model 2	Model 3	Model 4	Model 5
Constant	1.801*** (0.130)	1.364*** (0.155)	0.839*** (0.165)	7.566*** (0.260)	7.571*** (0.260)
Population: SPARK (reference: GenPop)	0.860*** (0.051)	0.813** (0.052)	0.771*** (0.051)	1.952*** (0.099)	1.936*** (0.099)
Education (reference: less than high school)					
High school	0.575*** (0.137)	0.556*** (0.137)	0.477*** (0.136)	1.116*** (0.264)	1.110*** (0.264)
Undergraduate	0.938*** (0.142)	0.907*** (0.142)	0.822*** (0.141)	1.701*** (0.274)	1.702*** (0.274)
Graduate	1.137*** (0.135)	1.102*** (0.135)	0.978*** (0.135)	2.703*** (0.260)	2.717*** (0.260)
Perceived importance of genetic knowledge	–	0.085*** (0.016)	–	–	–
Confidence in genetic knowledge	–	–	0.299*** (0.032)	–	–
Religious affiliation (reference: none)	–	–	–	−0.238*** (0.056)	–
Religious activity (reference: no religious affiliation)					
Religious, not actively practicing	–	–	–	–	−0.130* (0.040)
Religious, actively practicing	–	–	–	–	−0.358*** (0.065)
R^2	0.102	0.108	0.121	0.106	0.109
ΔR^2 from model 1	–	0.006	0.019	0.004	0.007
No. of observations	4,057	4,057	4,057	4,057	4,057

Each cell contains the unstandardized regression coefficient (standard error in parentheses), representing the change in Skills score associated with a one-unit increase in continuous predictors or the difference from the reference category for categorical predictors. R^2 indicates the proportion of variance in Skills scores explained by each model. ΔR^2 from model 1 shows the additional variance explained by adding predictors beyond the base model. $n = 4,057$. ***$p < 0.001$; **$p < 0.01$; *$p < 0.05$.

have both significantly positive and negative effects on GL scores specifically should be factored into more thoughtful science communication around genetics and genomics. We further suggest that our GL measure could be coupled with interventions to judge effectiveness of communication or used in clinical practice as a pre-test to help determine the best use of time and effort.

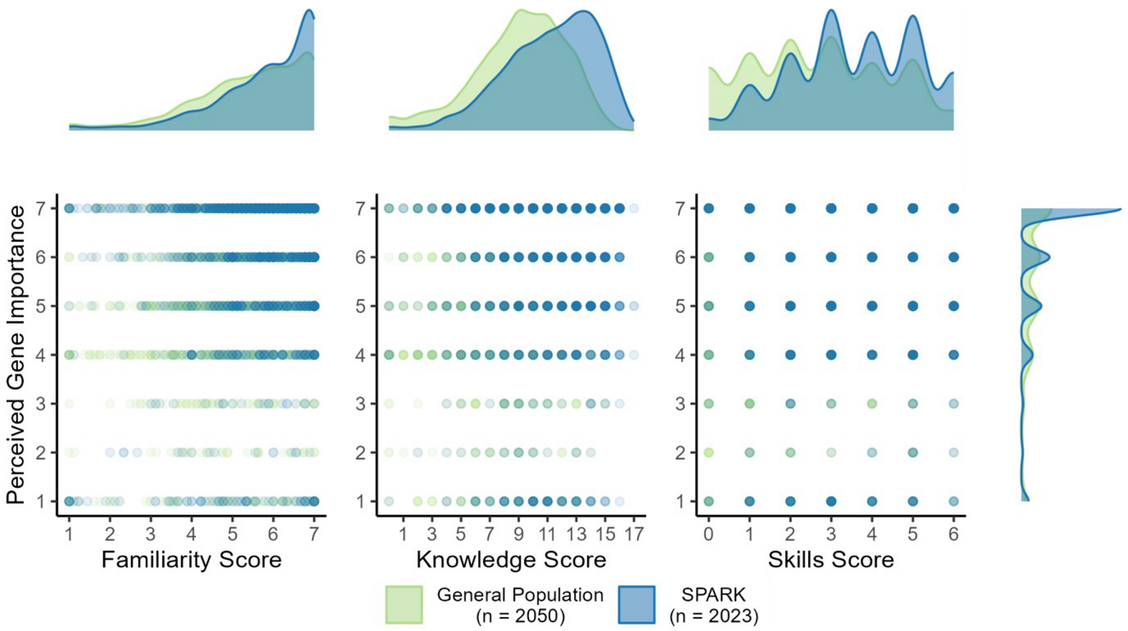

Figure 1. Scatterplots of perceived gene importance by each genetic literacy score and population
Density plots of each genetic literacy score by population are provided in the upper margin. The density plot of the perceived gene importance by population is provided in the right margin.

Table 6. Linear regression model results for each GL score by population, education, and belief in religion or science

	Familiarity score	Knowledge score	Skills score
Intercept	4.638*** (0.246), $p < 0.001$	8.253*** (0.578), $p < 0.001$	2.537*** (0.343), $p < 0.001$
Population—SPARK	0.274*** (0.081), $p < 0.001$	1.868*** (0.19), $p < 0.001$	0.561*** (0.113), $p < 0.001$
Education—high school	0.601* (0.255), $p = 0.019$	0.357 (0.599), $p = 0.552$	0.038 (0.356), $p = 0.914$
Education—undergraduate	0.874*** (0.259), $p < 0.001$	0.887 (0.608), $p = 0.145$	0.39 (0.362), $p = 0.281$
Education—graduate	0.92*** (0.248), $p < 0.001$	1.436* (0.583), $p = 0.014$	0.346 (0.346), $p = 0.318$
Belief in science	0.332*** (0.083), $p < 0.001$	1.018*** (0.196), $p < 0.001$	0.401*** (0.116), $p < 0.001$
n	857	857	857
R^2	0.075	0.198	0.068
Overall F statistic	13.884	42.104	12.438
Overall F p value	<0.001	<0.001	<0.001

Entries are coefficient (standard error), and asterisks indicate statistical significance. ***$p < 0.001$; **$p < 0.01$; *$p < 0.05$.

Perceptions of importance and confidence

In previous work, Kaphingst et al.[24] found an inverse relationship between health literacy and the perceived importance of genetic information, e.g., those with limited health literacy were more likely to think that genetic information was important. Our GALS did not demonstrate the same relationship; we found that those with higher GALS subscale scores also reported higher importance (Figure 1; Tables 3, 4, and 5). We also saw that SPARK participants had a higher average perceived importance of genetic information, as might be expected from those participating in a genetics research study. Similarly, the SPARK participants reported both more confidence in their own genetic knowledge and showed higher actual genetic knowledge than the GenPop sample.

Because our Familiarity measure is subjective knowledge, it fits expectations that we saw the largest proportion of the variance explained by confidence in genetic knowledge for our Familiarity scale than any of the variables tested, with an R^2 of 0.255. This held true after removing the effects of SPARK vs. GenPop or education level (Table 4). Confidence in genetic knowledge also showed the largest effect on our objective Knowledge scale, similarly explaining approximately 25% of the variance (R^2 of 0.256). While numerous studies have examined the confidence of healthcare providers related to genetics,[28–30] fewer have explored the confidence of general population or research study participants; our data suggest that this area could benefit from intervention and assessment in a manner that does not promote overconfidence without sufficient knowledge.[12,31]

Religious concepts show influence on the population's GL

Our findings are similar to those of Parrott et al.[20] and Fonseca et al.[31] in finding a significant but minor negative correlation between either subjective or objective knowledge and religiosity. Our study, however, differs from those and from Chapman et al.[5] in two ways: first, the difference of scores in all three subscales between self-declared religious and non-religious groups was significant only in the population of research participants and not in the general population. Second, we include the Skills section as a measure of knowledge comprehension and find that it acts similarly to the other scales in this case.

In comparing to the 2018 Wellcome/Gallup global study (https://cms.wellcome.org/sites/default/files/wellcome-global-monitor-2018.pdf), we found that both of our population samples reported levels of religious affiliation (76% GenPop and 66% SPARK) fairly similar to those of their North America participants (76%). When we asked for a choice between religion or science in the case of a disagreement, the large majority of both samples actually said the choice was "not applicable." However, of the minority who did make a selection (Table 2, "conflict between religion and science"), we saw a significant difference in those choosing religion in the GenPop (53%, similar to the Wellcome/Gallup figure of 58%) vs. the SPARK group (27%). This is consistent with the idea that those choosing to participate in a genetic research study generally may place a higher belief in science; along those lines, studies of the UK Biobank have concluded that selective participation biases both who chooses to participate and analyses based on the participants, including socio-behavioral phenotypes.[32,33] Specific to SPARK, Tafolla et al.[34] note that "advancing autism knowledge" and having more information were motivators for at least a subset of participants, again suggesting a higher relative value placed on science, which our study confirms. As with our other identity/belief tests, we saw higher GL scores in the subset of SPARK participants who chose either science or religion than in the GenPop sample (Figure S4).

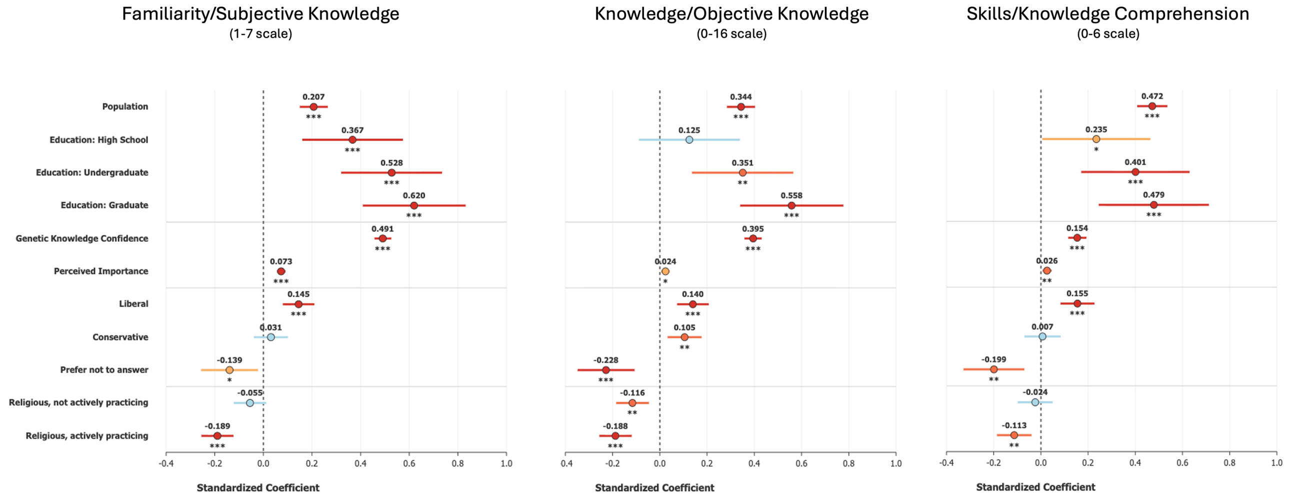

Figure 2. Forest plot of standardized regression coefficients β with 95% confidence intervals for Familiarity, Knowledge, and Skills, plotted by measure or category of measure

Points represent standardized coefficient estimates, and horizontal lines indicate 95% confidence intervals. Asterisks underneath each point indicate significance if present: ***$p < 0.001$ (red); **$p < 0.01$ (orange); *$p < 0.05$ (yellow); no asterisk, not significant (blue).

Political beliefs show a more complex influence on components of GL

As Heslop et al. found in their 2021 survey of political leaning and science communication (https://mediaengagement.org/research/communicating-science-across-political-divides/), Americans overall have a preference for "science communication that builds connections and encourages people to use science in their daily lives," in keeping with the construct of GL as application to one's own life. Our data for self-chosen political beliefs in the GenPop sample (33.7% conservative, 36.5% moderate, and 29.8% liberal) mirrored that seen in the 2021 Gallup poll of 12,416 Americans (https://news.gallup.com/poll/388988/political-ideology-steady-conservatives-moderates-tie.aspx), which found 36% self-described as conservative, 37% as moderate, and 25% as liberal. In contrast, the SPARK sample was 17.6% conservative, 28.5% moderate, and 40.6% liberal, with a larger proportion of participants choosing to not declare any belief on this scale. Our combined sample is biased by both those who chose to participate in SPARK and those who chose to take our survey in either group. With that said, our sample showed that "conservative" and "moderate" only significantly differ from each other on the Knowledge scale in our regression analyses, while declaring a "liberal" belief showed increases on all three of our subscales.

Limitations of the study

Our study has a few limitations in the scales used. For this GALS version, as we noted in Little et al.,[8] we adapted the Skills subscale from the original[7] because we wanted to shift the focus from breast cancer to autism, and we wanted to focus on complex genetics. This subscale is an important piece of the GALS because it measures comprehension, a dimension that is included in most definitions of GL but not included in almost any other currently used measure of GL.[3] Our current analyses clarified that the scores on this subscale were lower than we expected, possibly for two reasons: questions including double negatives may have played a role in low comprehension, and the new infographic we created (adapted from the cup-and-ball model of inheritance by Hoang et al.[35]) may have contained too much jargon. Future versions of our survey have re-worded both the questions and the infographic to be clearer and more understandable; we are also taking the measure through an extensive validation process. In addition, feedback from our SPARK participants spurred us to update language in our survey instrument about autism, including using person-first language and avoiding stereotypes.[36] Finally, we have only included samples from the US and note that previous studies found results on tests of genetic knowledge and attitudes vary in nuanced ways between samples from other countries.[37]

Conclusions

We suggest that the specifics of both religious beliefs and political belief here are less important than the observation that, consistent with cultural cognition theory,[11] group identities can dictate the patterns in which individuals selectively assess evidence. They can also dictate the most appropriate modes of science communication, as in the Heslop et al. (https://mediaengagement.org/research/communicating-science-across-political-divides/) US survey finding that some political belief groups favored communication through media professionals while others preferred face-to-face communication. Genetics providers and researchers interested in fostering general understanding of our field, as well as informed uptake of genetic/genomic services, should recognize that the deficit model of science communication—simply providing facts

to individuals and then expecting that they will accept scientific concepts because they only lacked understanding[27]—is incorrect and inapplicable to achieving their goals.

As noted in a recent review of considerations for integrating polygenic risk scores into clinical practice,[38] visual aids such as the "cup-and-ball" model we used in our Skills subscale,[35] fuzzy trace theory/gist approaches for communicating information,[39] or other decision support tools based on cognitive interviewing[40] are likely examples of science communication approaches that would be more effective in improving GL. Effective genetics communication also relies on building trust between scientists and communities,[11,41] which has remained strong over time both in the US (https://www.pewresearch.org/science/2024/11/14/public-trust-in-scientists-and-views-on-their-role-in-policymaking/) and globally.[42] As our data show that improving the confidence in one's own genetic knowledge is, after education, the largest positive predictor of performance on our scales, we therefore must stress the importance of not undermining trust in science or scientists with misinformation about genetics or other topics.

Data and code availability

The raw data utilized in this study were derived from a larger research project. The dataset will be publicly available after the initial articles reporting on the collected data are published. Summary statistics are published in Little et al.[8] Until then, the dataset can be accessed by contacting the corresponding author (chris.gunter@nih.gov) upon reasonable request. Code for statistics and figures can be accessed at https://github.com/ChrisG-EMU/Ramirez-Renta-et-al-paper-genetic-literacy.

Acknowledgments

This project was funded by a SPARK Research Match grant (RM0149) and the National Institutes of Health: National Human Genome Research Institute Intramural Research Program (HG200410-01). The contributions of the NIH authors are considered Works of the United States Government. The findings and conclusions presented in this paper are those of the authors and do not necessarily reflect the views of the NIH or the US Department of Health and Human Services. We thank Daisy Yu of The Emmes Company for statistical assistance in the early stages of GALS, and the Gunter and Persky labs at NHGRI for suggestions throughout.

Author contributions

Conception and design of experiments, G.M.R.R., I.D.L., L.M.K., and C.G.; data analysis, G.M.R.R., I.D.L., A.J.H., K.L.F., J.B., J.L., and C.G. All authors have reviewed and approved the final version of the manuscript.

Declaration of interests

The authors declare no competing interests.

Declaration of generative AI and AI-assisted technologies in the writing process

During the preparation of this work, the authors used Claude v.4.1 Opus in order to provide code for standardizing coefficients and to make Figure 2. After using this tool/service, the authors reviewed and edited the content as needed and take full responsibility for the content of the publication.

Supplemental information

Supplemental information can be found online at https://doi.org/10.1016/j.ajhg.2025.11.014.

Web resources

Claude Opus 4.5, https://www.anthropic.com/claude/opus

Gallup poll on political ideology, https://news.gallup.com/poll/388988/political-ideology-steady-conservatives-moderates-tie.aspx

Pew Research Center report on public trust in scientists, https://www.pewresearch.org/science/2024/11/14/public-trust-in-scientists-and-views-on-their-role-in-policymaking/

Report by Heslop et al., https://mediaengagement.org/research/communicating-science-across-political-divides/

The R Project for Statistical Computing, https://www.r-project.org/

Wellcome Global Monitor 2018, https://cms.wellcome.org/sites/default/files/wellcome-global-monitor-2018.pdf

Received: July 17, 2025
Accepted: November 24, 2025
Published: December 22, 2025

References

1. Halbisen, A.L., and Lu, C.Y. (2023). Trends in Availability of Genetic Tests in the United States, 2012–2022. J. Pers. Med. *13*, 638.
2. Hurle, B., Citrin, T., Jenkins, J.F., Kaphingst, K.A., Lamb, N., Roseman, J.E., and Bonham, V.L. (2013). What does it mean to be genomically literate?: National Human Genome Research Institute Meeting Report. Genet. Med. *15*, 658–663. https://doi.org/10.1038/gim.2013.14.
3. Daly, B.M., and Kaphingst, K.A. (2023). Variability in conceptualizations and measurement of genetic literacy. PEC Innov. *2*, 100147. https://doi.org/10.1016/j.pecinn.2023.100147.
4. Erby, L.H., Roter, D., Larson, S., and Cho, J. (2008). The rapid estimate of adult literacy in genetics (REAL-G): A means to assess literacy deficits in the context of genetics. Am. J. Med. Genet. *146A*, 174–181. https://doi.org/10.1002/ajmg.a.32068.
5. Chapman, R., Likhanov, M., Selita, F., Zakharov, I., Smith-Woolley, E., and Kovas, Y. (2019). New literacy challenge for the twenty-first century: genetic knowledge is poor even among well educated. J. Commun. Genet. *10*, 73–84. https://doi.org/10.1007/s12687-018-0363-7.
6. Linderman, M.D., Suckiel, S.A., Thompson, N., Weiss, D.J., Roberts, J.S., and Green, R.C. (2021). Development and Validation of a Comprehensive Genomics Knowledge Scale. Public Health Genom. *24*, 291–303. https://doi.org/10.1159/000515006.

7. Abrams, L.R., McBride, C.M., Hooker, G.W., Cappella, J.N., and Koehly, L.M. (2015). The many facets of genetic literacy: Assessing the scalability of multiple measures for broad use in survey research. PLoS One *10*, e0141532. https://doi.org/10.1371/journal.pone.0141532.
8. Little, I.D., Koehly, L.M., and Gunter, C. (2022). Understanding changes in genetic literacy over time and in genetic research participants. Am. J. Hum. Genet. *109*, 2141–2151. https://doi.org/10.1016/j.ajhg.2022.11.005.
9. Kahan, D.M., Braman, D., Slovic, P., Gastil, J., and Cohen, G. (2009). Cultural cognition of the risks and benefits of nanotechnology. Nat. Nanotechnol. *4*, 87–90. https://doi.org/10.1038/nnano.2008.341.
10. Kaanders, P., Sepulveda, P., Folke, T., Ortoleva, P., and De Martino, B. (2022). Humans actively sample evidence to support prior beliefs. eLife *11*, e71768. https://doi.org/10.7554/eLife.71768.
11. Kahan, D. (2015). What is the "science of science communication". J. Sci. Commun. *14*, 12. https://doi.org/10.22323/2.14030404.
12. Lackner, S., Francisco, F., Mendonça, C., Mata, A., and Gonçalves-Sá, J. (2023). Intermediate levels of scientific knowledge are associated with overconfidence and negative attitudes towards science. Nat. Hum. Behav. *7*, 1490–1501. https://doi.org/10.1038/s41562-023-01677-8.
13. Carere, D.A., Kraft, P., Kaphingst, K.A., Roberts, J.S., and Green, R.C. (2016). Consumers report lower confidence in their genetics knowledge following direct-to-consumer personal genomic testing. Genet. Med. *18*, 65–72. https://doi.org/10.1038/gim.2015.34.
14. Adams, E.J., Asad, S., Reinbolt, R., Collier, K.A., Abdel-Rasoul, M., Gillespie, S., Chen, J.L., Cherian, M.A., Noonan, A.M., Sardesai, S., et al. (2020). Metastatic breast cancer patient perceptions of somatic tumor genomic testing. BMC Cancer *20*, 389. https://doi.org/10.1186/s12885-020-06905-2.
15. McConkie-Rosell, A., Spillmann, R.C., Schoch, K., Sullivan, J.A., Walley, N., McDonald, M., Undiagnosed Diseases Network, Hooper, S.R., and Shashi, V. (2023). Unraveling non-participation in genomic research: A complex interplay of barriers, facilitators, and sociocultural factors. J. Genet. Couns. *32*, 993–1008. https://doi.org/10.1002/jgc4.1707.
16. Gligoric, V., van Kleef, G.A., and Rutjens, B.T. (2022). Social evaluations of scientific occupations. Sci. Rep. *12*, 18339. https://doi.org/10.1038/s41598-022-23197-7.
17. Gollust, S.E., Gray, S.W., Carere, D.A., Koenig, B.A., Lehmann, L.S., McGuire, A.L., Sharp, R.R., Spector-Bagdady, K., Wang, N.A., Green, R.C., et al. (2017). Consumer Perspectives on Access to Direct-to-Consumer Genetic Testing: Role of Demographic Factors and the Testing Experience. Milbank Q. *95*, 291–318. https://doi.org/10.1111/1468-0009.12262.
18. Botoseneanu, A., Alexander, J.A., and Banaszak-Holl, J. (2011). To test or not to test? The role of attitudes, knowledge, and religious involvement among U.S. adults on intent-to-obtain adult genetic testing. Health Educ. Behav. *38*, 617–628. https://doi.org/10.1177/1090198110389711.
19. Allum, N., Sibley, E., Sturgis, P., and Stoneman, P. (2014). Religious beliefs, knowledge about science and attitudes towards medical genetics. Public Underst. Sci. *23*, 833–849. https://doi.org/10.1177/0963662513492485.
20. Parrott, R., Silk, K., Raup Krieger, J., Harris, T., and Condit, C. (2004). Behavioral health outcomes associated with religious faith and media exposure about human genetics. Health Commun. *16*, 29–45. https://doi.org/10.1207/S15327027HC1601_3.
21. Middleton, A., Costa, A., Milne, R., Patch, C., Robarts, L., Tomlin, B., Danson, M., Henriques, S., Atutornu, J., Aidid, U., et al. (2023). The legacy of language: What we say, and what people hear, when we talk about genomics. HGG Adv. *4*, 100231. https://doi.org/10.1016/j.xhgg.2023.100231.
22. Simis, M.J., Madden, H., Cacciatore, M.A., and Yeo, S.K. (2016). The lure of rationality: Why does the deficit model persist in science communication? Public Underst. Sci. *25*, 400–414. https://doi.org/10.1177/0963662516629749.
23. Zajic, M.C., and Gudknecht, J. (2024). Person- and identity-first language in autism research: A systematic analysis of abstracts from 11 autism journals. Autism *28*, 2445–2461. https://doi.org/10.1177/13623613241241202.
24. Kaphingst, K.A., Blanchard, M., Milam, L., Pokharel, M., Elrick, A., and Goodman, M.S. (2016). Relationships Between Health Literacy and Genomics-Related Knowledge, Self-Efficacy, Perceived Importance, and Communication in a Medically Underserved Population. J. Health Commun. *21*, 58–68. https://doi.org/10.1080/10810730.2016.1144661.
25. García-Pérez, M.A. (2023). Use and misuse of corrections for multiple testing. Methods Psychol. *8*, 100120. https://doi.org/10.1016/j.metip.2023.100120.
26. Cohen, J. (1990). Things I have learned (so far). Am. Psychol. *45*, 1304–1312. https://doi.org/10.1037/0003-066X.45.12.1304.
27. Drummond, C., and Fischhoff, B. (2017). Individuals with greater science literacy and education have more polarized beliefs on controversial science topics. Proc. Natl. Acad. Sci. USA *114*, 9587–9592. https://doi.org/10.1073/pnas.1704882114.
28. Mir, Z.M., Fei, L.Y.N., McKeown, S., Dinchong, R., Cofie, N., Dalgarno, N., Rusnak, A., Cheifetz, R.E., and Merchant, S.J. (2024). Knowledge, perceptions, attitudes, and barriers pertaining to genetic literacy among surgeons: a scoping review. Can. J. Surg. *67*, E118–E127. https://doi.org/10.1503/cjs.001523.
29. East, K.M., Cochran, M.E., Kelley, W.V., Greve, V., Finnila, C.R., Coleman, T., Jennings, M., Alexander, L., Rahn, E.J., Danila, M.I., et al. (2022). Education and Training of Non-Genetics Providers on the Return of Genome Sequencing Results in a NICU Setting. J. Pers. Med. *12*, 405. https://doi.org/10.3390/jpm12030405.
30. Gates, R.W., Hudgins, L., and Huffman, L.C. (2022). Medical genetics education for pediatrics residents: A brief report. Genet. Med. *24*, 2408–2412. https://doi.org/10.1016/j.gim.2022.08.003.
31. Fonseca, C., Pettitt, J., Woollard, A., Rutherford, A., Bickmore, W., Ferguson-Smith, A., and Hurst, L.D. (2023). People with more extreme attitudes towards science have self-confidence in their understanding of science, even if this is not justified. PLoS Biol. *21*, e3001915. https://doi.org/10.1371/journal.pbio.3001915.
32. Battram, T., Gaunt, T.R., Relton, C.L., Timpson, N.J., and Hemani, G. (2022). A comparison of the genes and genesets identified by GWAS and EWAS of fifteen complex traits. Nat. Commun. *13*, 7816. https://doi.org/10.1038/s41467-022-35037-3.
33. Schoeler, T., Speed, D., Porcu, E., Pirastu, N., Pingault, J.B., and Kutalik, Z. (2023). Participation bias in the UK Biobank distorts genetic associations and downstream analyses. Nat.

Hum. Behav. *7*, 1216–1227. https://doi.org/10.1038/s41562-023-01579-9.

34. Tafolla, M., Amador, R., Oyeyemi, M.J., Algaze, Z., Pandey, J., Goin-Kochel, R.P., Sarver, D.E., and Gulsrud, A. (2024). Barriers, motivators and strategies to increase participation in genetic research among Asian and Black families of autistic individuals. J. Community Genet. *15*, 559–572. https://doi.org/10.1007/s12687-024-00724-9.
35. Hoang, N., Cytrynbaum, C., and Scherer, S.W. (2018). Communicating complex genomic information: A counselling approach derived from research experience with Autism Spectrum Disorder. Patient Educ. Couns. *101*, 352–361. https://doi.org/10.1016/j.pec.2017.07.029.
36. Bottema-Beutel, K., Kapp, S.K., Lester, J.N., Sasson, N.J., and Hand, B.N. (2021). Avoiding Ableist Language: Suggestions for Autism Researchers. Autism Adulthood. *3*, 18–29. https://doi.org/10.1089/aut.2020.0014.
37. Likhanov, M., Zakharov, I., Awofala, A., Ogundele, O., Selita, F., Kovas, Y., and Chapman, R. (2023). Attitudes towards genetic testing: The role of genetic literacy, motivated cognition, and socio-demographic characteristics. PLoS One *18*, e0293187. https://doi.org/10.1371/journal.pone.0293187.
38. Sanderson, S.C., and Inouye, M. (2025). Psychological and behavioural considerations for integrating polygenic risk scores for disease into clinical practice. Nat. Hum. Behav. *9*, 1098–1106. https://doi.org/10.1038/s41562-025-02200-x.
39. Wolfe, C.R., Reyna, V.F., Widmer, C.L., Cedillos, E.M., Fisher, C.R., Brust-Renck, P.G., and Weil, A.M. (2015). Efficacy of a web-based intelligent tutoring system for communicating genetic risk of breast cancer: a fuzzy-trace theory approach. Med. Decis. Mak. *35*, 46–59. https://doi.org/10.1177/0272989X14535983.
40. Bombard, Y., Clausen, M., Mighton, C., Carlsson, L., Casalino, S., Glogowski, E., Schrader, K., Evans, M., Scheer, A., Baxter, N., et al. (2018). The Genomics ADvISER: Development and usability testing of a decision aid for the selection of incidental sequencing results. Eur. J. Hum. Genet. *26*, 984–995. https://doi.org/10.1038/s41431-018-0144-0.
41. Besley, J.C., Lee, N.M., and Pressgrove, G. (2020). Reassessing the Variables Used to Measure Public Perceptions of Scientists. Sci. Commun. *43*, 3–32. https://doi.org/10.1177/1075547020949547.
42. Cologna, V., Mede, N.G., Berger, S., Besley, J., Brick, C., Joubert, M., Maibach, E.W., Mihelj, S., Oreskes, N., Schäfer, M.S., et al. (2025). Trust in scientists and their role in society across 68 countries. Nat. Hum. Behav. *9*, 713–730. https://doi.org/10.1038/s41562-024-02090-5.

The American Journal of Human Genetics, Volume *113*

Supplemental information

Interaction of identity and beliefs

with genetic literacy

Gabriela M. Ramírez Renta, India D. Little, Laura M. Koehly, Anna J. Hilliard, Kaylee L. Foor, Jessica Butts, Jordan Lundeen, and Chris Gunter

Supplemental Figures and Legends

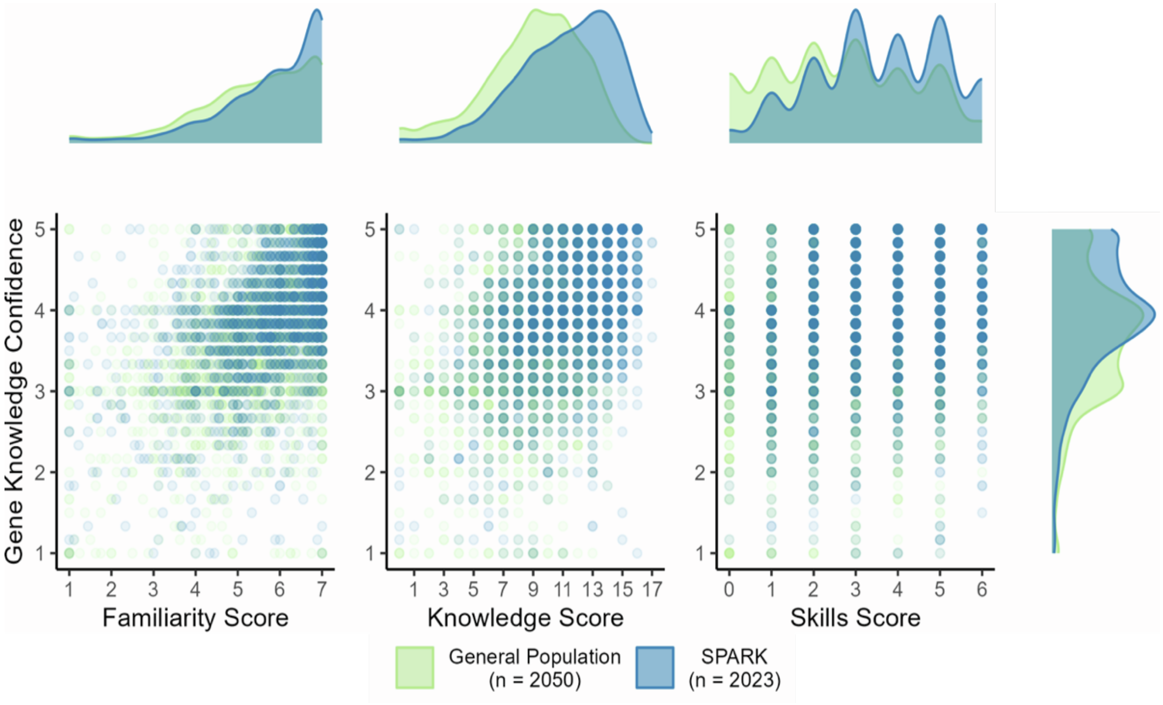

Figure S1: Scatter plots of genetic knowledge confidence by each genetic literacy score and population. Density plots of each genetic literacy score by population are provided in the upper margin. The density plot of the genetic knowledge confidence by population is provided in the right margin.

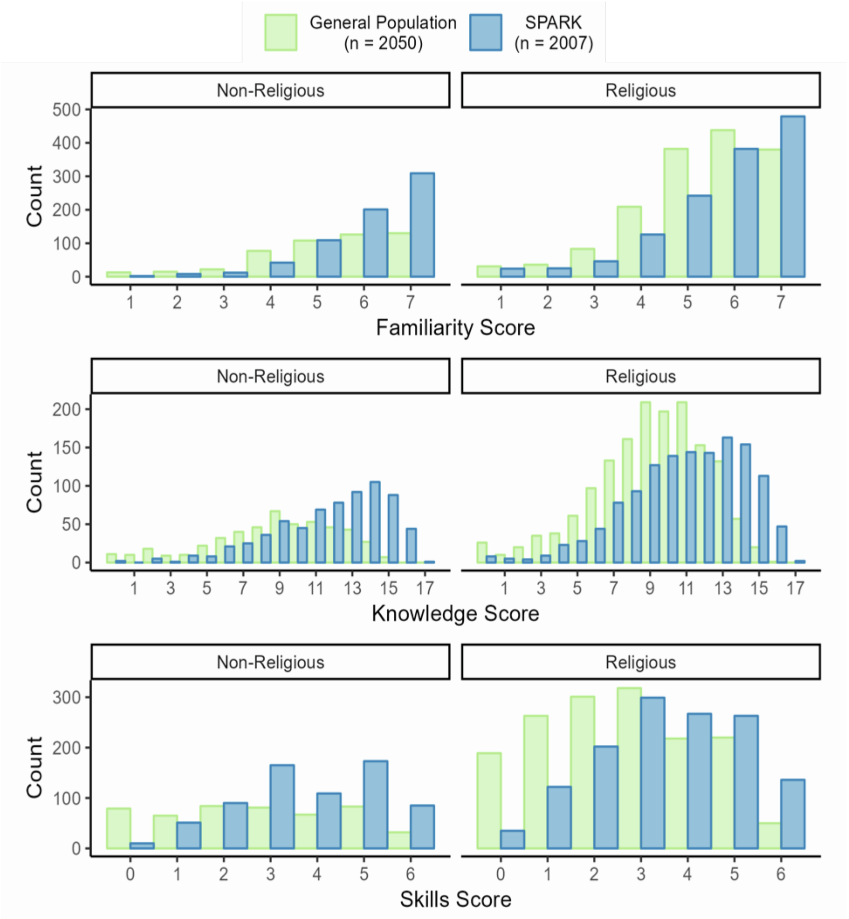

Figure S2: Histogram plots of each genetic literacy score by religious affiliation (Religious vs. Non-Religious) and population.

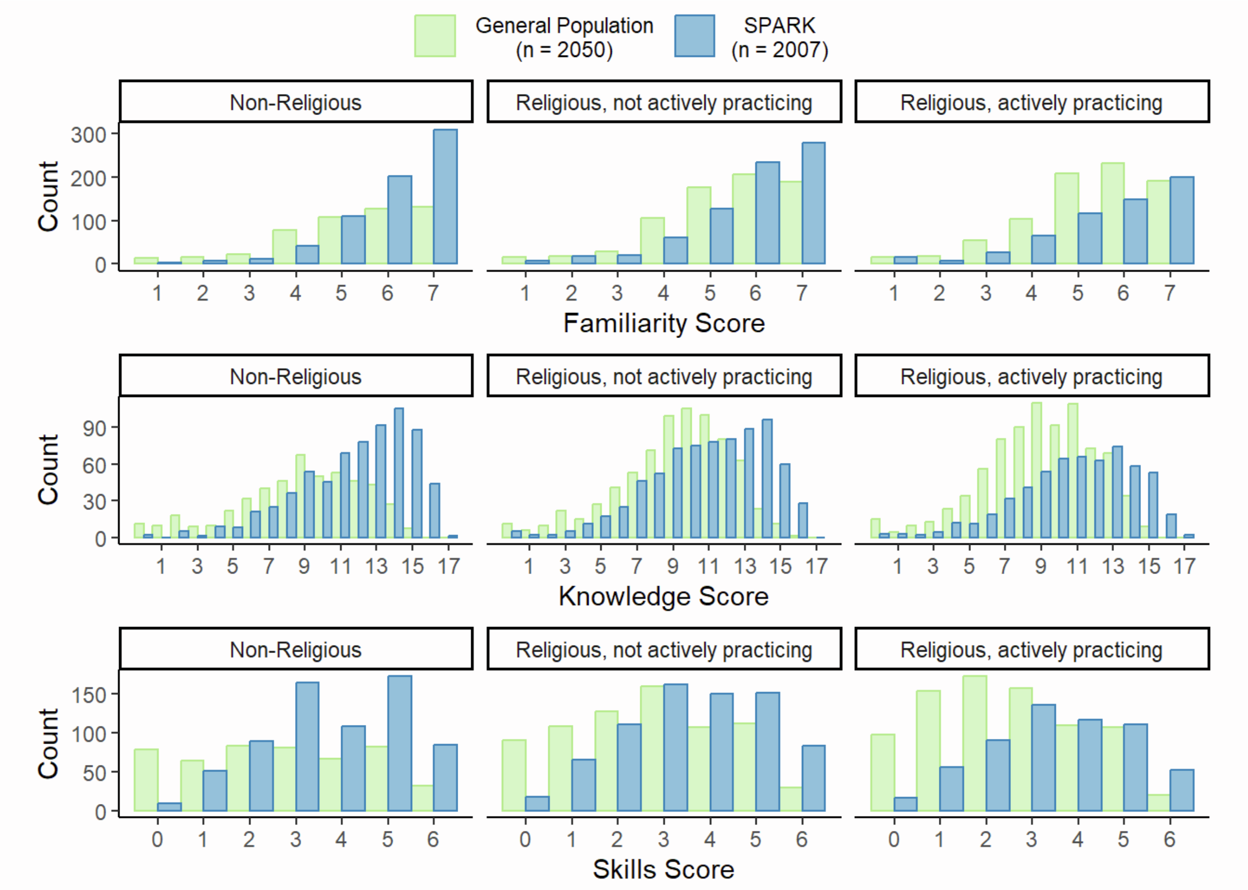

Figure S3: Histogram plots of each genetic literacy score by religiosity (Non-Religious vs. Religious, not actively practicing vs. Religious, actively practicing) and population.

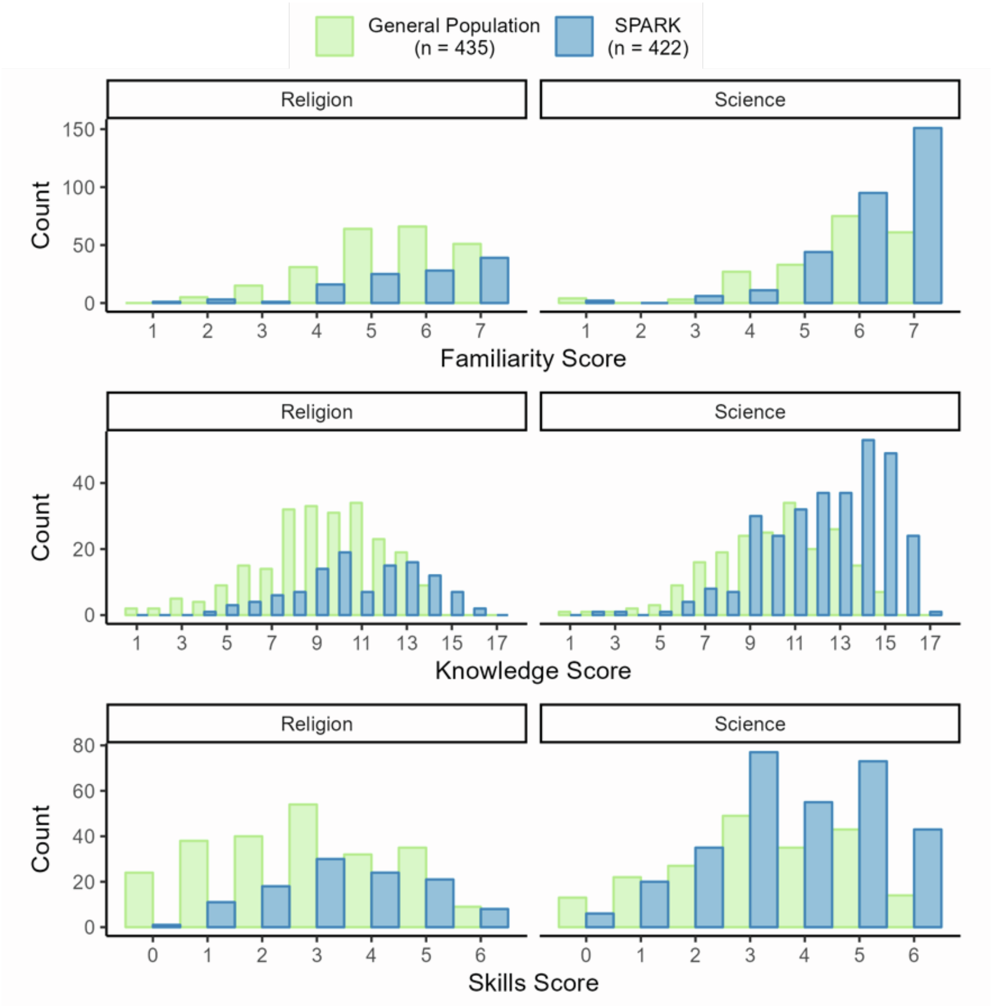

Figure S4. Histogram plots of each genetic literacy score by belief in Religion or Science (Religion vs. Science) and population.

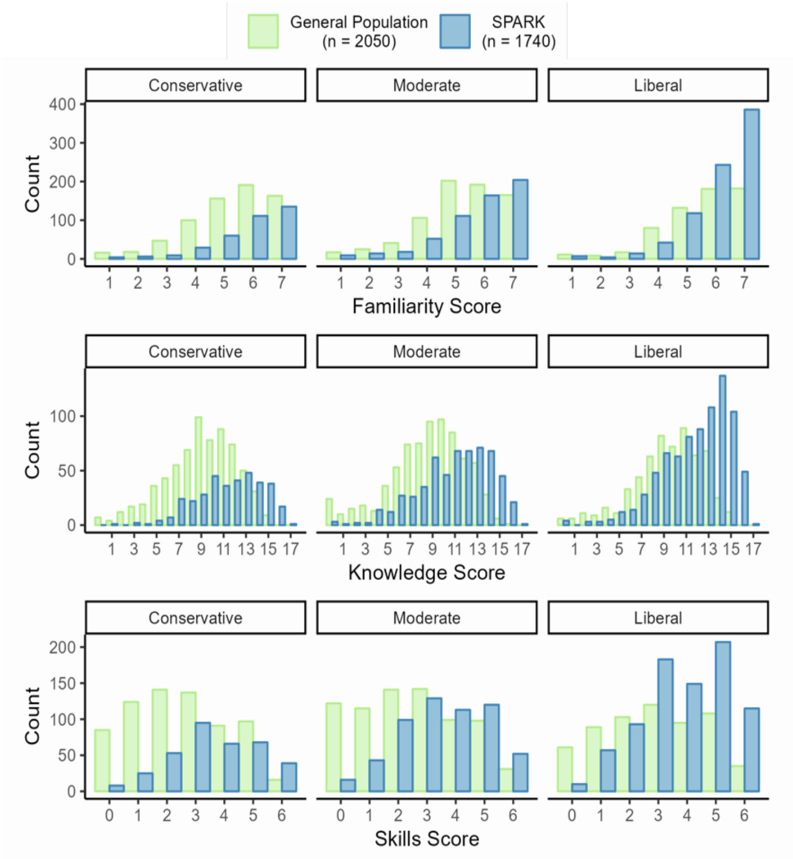

Figure S5. Histogram plots of each genetic literacy score by political belief and population.

Supplemental Tables

All tables showing linear regression models are formatted similarly: Each cell contains the unstandardized regression coefficient (standard error in parentheses), representing the change in GL subscale score associated with a one-unit increase in continuous predictors or the difference from the reference category for categorical predictors. R^2 indicates the proportion of variance in familiarity scores explained by each model. n = 4,057 unless otherwise indicated.

*** $p < 0.001$; ** $p < 0.01$; * $p < 0.05$.

	**Familiarity Score**	**Knowledge Score**	**Skills Score**
**Intercept**	3.508 *** (0.122)<br>p: <0.001	5.901 *** (0.297)<br>p: <0.001	1.364 *** (0.155)<br>p: <0.001
**Population - SPARK**	0.234 *** (0.041)<br>p: <0.001	1.856 *** (0.099)<br>p: <0.001	0.813 *** (0.052)<br>p: <0.001
**Education - High School**	0.615 *** (0.108)<br>p: <0.001	1.012 *** (0.262)<br>p: <0.001	0.556 *** (0.137)<br>p: <0.001
**Education - Undergraduate**	0.833 *** (0.112)<br>p: <0.001	1.532 *** (0.272)<br>p: <0.001	0.907 *** (0.142)<br>p: <0.001
**Education - Graduate**	1.069 *** (0.106)<br>p: <0.001	2.517 *** (0.258)<br>p: <0.001	1.102 *** (0.135)<br>p: <0.001
**Perceived Importance Genetic Knowledge**	0.196 *** (0.013)<br>p: <0.001	0.265 *** (0.031)<br>p: <0.001	0.085 *** (0.016)<br>p: <0.001
n	4057	4057	4057
R^2	0.114	0.166	0.108
Overall F statistic	104.056	161.305	98.235
Overall F p value	<0.001	<0.001	<0.001

*** $p < 0.001$; ** $p < 0.01$; * $p < 0.05$.

Table S1: Linear regression model results for each GL score by population, education, and perceived gene importance. Entries are the "coefficient (standard error)," and stars indicate statistical significance.

	Familiarity Score	Knowledge Score	Skills Score
**Intercept**	2.094 *** (0.12)<br>p: <0.001	2.77 *** (0.301)<br>p: <0.001	0.839 *** (0.165)<br>p: <0.001
**Population - SPARK**	0.12 ** (0.037)<br>p: 0.001	1.589 *** (0.094)<br>p: <0.001	0.771 *** (0.051)<br>p: <0.001
**Education - High School**	0.412 *** (0.099)<br>p: <0.001	0.613 * (0.248)<br>p: 0.013	0.477 *** (0.136)<br>p: <0.001
**Education - Undergraduate**	0.612 *** (0.103)<br>p: <0.001	1.087 *** (0.258)<br>p: <0.001	0.822 *** (0.141)<br>p: <0.001
**Education - Graduate**	0.749 *** (0.098)<br>p: <0.001	1.882 *** (0.246)<br>p: <0.001	0.978 *** (0.135)<br>p: <0.001
**Confidence in Genetic Knowledge**	0.753 *** (0.023)<br>p: <0.001	1.396 *** (0.058)<br>p: <0.001	0.299 *** (0.032)<br>p: <0.001
n	4057	4057	4057
R^2	0.255	0.256	0.121
Overall *F* statistic	277.333	279.426	111.647
Overall *F* *p* value	<0.001	<0.001	<0.001

*** $p < 0.001$; ** $p < 0.01$; * $p < 0.05$.

Table S2: Linear regression model results for each GL score by population, education, and genetic knowledge confidence. Entries are the "coefficient (standard error)," and stars indicate statistical significance.

	Familiarity Score	Knowledge Score	Skills Score
**Intercept**	4.652 *** (0.109)<br>p: <0.001	7.566 *** (0.26)<br>p: <0.001	1.951 *** (0.135)<br>p: <0.001
**Population - SPARK**	0.32 *** (0.041)<br>p: <0.001	1.952 *** (0.099)<br>p: <0.001	0.834 *** (0.051)<br>p: <0.001
**Education - High School**	0.679 *** (0.111)<br>p: <0.001	1.116 *** (0.264)<br>p: <0.001	0.598 *** (0.137)<br>p: <0.001
**Education - Undergraduate**	0.936 *** (0.115)<br>p: <0.001	1.701 *** (0.274)<br>p: <0.001	0.975 *** (0.142)<br>p: <0.001
**Education – Graduate**	1.184 *** (0.109)<br>p: <0.001	2.703 *** (0.26)<br>p: <0.001	1.175 *** (0.135)<br>p: <0.001
**Religious**	-0.210 *** (0.046)<br>p: <0.001	-0.472 *** (0.109)<br>p: <0.001	-0.238 *** (0.056)<br>p: <0.001
n	4057	4057	4057
R^2	0.068	0.155	0.106
Overall *F* statistic	59.563	148.962	96.231
Overall *F p* value	<0.001	<0.001	<0.001

*** $p < 0.001$; ** $p < 0.01$; * $p < 0.05$.

Table S3: Linear regression model results for each GL score by population, education, and religious affiliation. Entries are the “coefficient (standard error),” and stars indicate statistical significance.

	Familiarity Score	Knowledge Score	Skills Score
**Intercept**	4.656 *** (0.109)<br>p: <0.001	7.571 *** (0.26)<br>p: <0.001	1.956 *** (0.135)<br>p: <0.001
**Population - SPARK**	0.308 *** (0.041)<br>p: <0.001	1.936 *** (0.099)<br>p: <0.001	0.819 *** (0.051)<br>p: <0.001
**Education - High School**	0.674 *** (0.111)<br>p: <0.001	1.110 *** (0.264)<br>p: <0.001	0.592 *** (0.137)<br>p: <0.001
**Education - Undergraduate**	0.937 *** (0.115)<br>p: <0.001	1.702 *** (0.274)<br>p: <0.001	0.976 *** (0.142)<br>p: <0.001
**Education – Graduate**	1.195 *** (0.109)<br>p: <0.001	2.717 *** (0.26)<br>p: <0.001	1.1789 *** (0.135)<br>p: <0.001
**Religious, not actively practicing**	-0.125 * (0.051)<br>p: 0.015	-0.363 ** (0.122)<br>p: 0.003	-0.130 * (0.04)<br>p: 0.04
**Religious, actively practicing**	-0.306 *** (0.052)<br>p: <0.001	-0.593 *** (0.125)<br>p: <0.001	-0.358 *** (0.065)<br>p: <0.001
n	4057	4057	4057
R^2	0.072	0.156	0.109
Overall *F* statistic	52.092	124.871	82.827
Overall *F p* value	<0.001	<0.001	<0.001

*** $p < 0.001$; ** $p < 0.01$; * $p < 0.05$.

Table S4: Linear regression model results for each GL score by population, education, and religiosity. Beliefs are regressed against “Not religious.” Entries are the “coefficient (standard error),” and stars indicate statistical significance.

	Familiarity Score	Knowledge Score	Skills Score
**Intercept**	4.407 *** (0.11)<br>p: <0.001	6.963 *** (0.263)<br>p: <0.001	1.71 *** (0.138)<br>p: <0.001
**Population - SPARK**	0.374 *** (0.043)<br>p: <0.001	2.151 *** (0.102)<br>p: <0.001	0.884 *** (0.054)<br>p: <0.001
**Education - High School**	0.703 *** (0.114)<br>p: <0.001	1.151 *** (0.271)<br>p: <0.001	0.622 *** (0.143)<br>p: <0.001
**Education - Undergraduate**	0.933 *** (0.118)<br>p: <0.001	1.663 *** (0.281)<br>p: <0.001	0.925 *** (0.148)<br>p: <0.001
**Education - Graduate**	1.132 *** (0.112)<br>p: <0.001	2.533 *** (0.267)<br>p: <0.001	1.126 *** (0.14)<br>p: <0.001
**Political Belief - Conservative**	0.031 (0.053)<br>p: 0.561	0.329 ** (0.127)<br>p: 0.01	-0.016 (0.067)<br>p: 0.812
**Political Belief - Liberal**	0.301 *** (0.05)<br>p: <0.001	0.68 *** (0.118)<br>p: <0.001	0.295 *** (0.062)<br>p: <0.001
n	3790	3790	3790
R^2	0.083	0.178	0.12
Overall *F* statistic	57.166	136.742	86.21
Overall *F* *p* value	<0.001	<0.001	<0.001

*** $p < 0.001$; ** $p < 0.01$; * $p < 0.05$.

Table S5: Linear regression model results for each GL score by population, education, and political belief. Beliefs are regressed against "Political belief - Moderate." Entries are the "coefficient (standard error)," and stars indicate statistical significance.

Tukey Comparisons of Means for Political Belief

	Familiarity Score	Knowledge Score	Skills Score
**Conservative – Moderate**	0.031 (0.05)<br>p: 0.830	0.329 * (0.13)<br>p: 0.026	-0.016 (0.07)<br>p: 0.969
**Liberal – Moderate**	0.301 *** (0.05)<br>p: <0.0001	0.680 *** (0.12)<br>p: <0.001	0.294 *** (0.06)<br>p: <0.00001
**Liberal – Conservative**	0.270 *** (0.05)<br>p: <0.0001	0.351 * (0.13)<br>p: 0.016	0.311 *** (0.07)<br>p: <0.00001

Table S6: Tukey pairwise comparison of means for each GL score by population, education, and political belief. Entries are the "coefficient (standard error)" and stars equal indicate statistical significance (*** $p < 0.001$; ** $p < 0.01$; * $p < 0.05$).

Interactions of Religious Affiliation, Religiosity, and Political Belief

We are unable to include both religious affiliation and religiosity in the same model because these two variables have overlapping definitions for "Non-Religious," so we looked at religiosity and political belief and then religious affiliation and political belief separately.

Religiosity and Political Belief: Tables S7-9

For all three genetic literacy scores, there does not seem to be much information gained by adding religiosity to the model that already includes population, education, and political belief or by adding political belief to the model that already includes population, education, and religiosity. The absolute changes in R^2 are all less than 1%.

Familiarity Score

	Full Model	Political Belief Only	Religiosity Only
**Intercept**	4.507 *** (0.114) p: <0.001	4.407 *** (0.110) p: <0.001	4.630 *** (0.112) p: <0.001
**Population - SPARK**	0.354 *** (0.043) p: <0.001	0.374 *** (0.043) p: <0.001	0.382 *** (0.043) p: <0.001
**Education - High School**	0.715 *** (0.114) p: <0.001	0.703 *** (0.114) p: <0.001	0.715 *** (0.114) p: <0.001
**Education - Undergraduate**	0.960 *** (0.118) p: <0.001	0.933 *** (0.118) p: <0.001	0.977 *** (0.118) p: <0.001
**Education - Graduate**	1.171 *** (0.112) p: <0.001	1.132 *** (0.112) p: <0.001	1.205 *** (0.112) p: <0.001
**Religiosity - Religious, actively practicing**	-0.246 *** (0.056) p: <0.001	--	-0.302 *** (0.054) p: <0.001
**Religiosity - Religious, not actively practicing**	-0.090 (0.053) p: 0.086	--	-0.127 * (0.052) p: 0.015
**Political Belief - Conservative**	0.071 (0.054) p: 0.188	0.031 (0.053) p: 0.561	--
**Political Belief - Liberal**	0.274 *** (0.050) p: <0.001	0.301 *** (0.050) p: <0.001	--
n	3790	3790	3790
R^2	0.088	0.083	0.081
Overall *F* statistic	45.661	57.166	55.212
Overall *F p* value	<0.001	<0.001	<0.001

*** $p < 0.001$; ** $p < 0.01$; * $p < 0.05$.

Table S7: Linear regression model results for the Familiarity GL score by both religiosity and political belief; only political belief; or only religiosity. Entries are the “coefficient (standard error),” and stars indicate statistical significance.

Knowledge Score

	Full Model	Political Belief Only	Religiosity Only
**Intercept**	7.226 *** (0.272) p: <0.001	6.963 *** (0.263) p: <0.001	7.539 *** (0.266) p: <0.001
**Population - SPARK**	2.111 *** (0.102) p: <0.001	2.151 *** (0.102) p: <0.001	2.148 *** (0.102) p: <0.001
**Education - High School**	1.187 *** (0.271) p: <0.001	1.151 *** (0.271) p: <0.001	1.200 *** (0.272) p: <0.001
**Education - Undergraduate**	1.730 *** (0.281) p: <0.001	1.663 *** (0.281) p: <0.001	1.782 *** (0.282) p: <0.001
**Education - Graduate**	2.619 *** (0.267) p: <0.001	2.533 *** (0.267) p: <0.001	2.702 *** (0.268) p: <0.001
**Religiosity - Religious, actively practicing**	-0.534 *** (0.132) p: <0.001	--	-0.592 *** (0.128) p: <0.001
**Religiosity - Religious, not actively practicing**	-0.329 ** (0.125) p: 0.009	--	-0.381 ** (0.124) p: 0.002
**Political Belief - Conservative**	0.409 ** (0.129) p: 0.001	0.329 ** (0.127) p: 0.010	--
**Political Belief - Liberal**	0.615 *** (0.119) p: <0.001	0.680 *** (0.118) p: <0.001	--
n	3790	3790	3790
R^2	0.182	0.178	0.176
Overall *F* statistic	105.002	136.742	134.476
Overall *F p* value	<0.001	<0.001	<0.001

*** $p < 0.001$; ** $p < 0.01$; * $p < 0.05$.

Table S8: Linear regression model results for Knowledge GL score by both religiosity and political belief; only political belief; or only religiosity. Entries are the "coefficient (standard error)," and stars indicate statistical significance.

Skills Score

	Full Model	Political Belief Only	Religiosity Only
**Intercept**	1.845 *** (0.143)<br>p: <0.001	1.710 *** (0.138)<br>p: <0.001	1.957 *** (0.140)<br>p: <0.001
**Population - SPARK**	0.859 *** (0.054)<br>p: <0.001	0.884 *** (0.054)<br>p: <0.001	0.889 *** (0.053)<br>p: <0.001
**Education - High School**	0.638 *** (0.142)<br>p: <0.001	0.622 *** (0.143)<br>p: <0.001	0.637 *** (0.143)<br>p: <0.001
**Education - Undergraduate**	0.960 *** (0.148)<br>p: <0.001	0.925 *** (0.148)<br>p: <0.001	0.975 *** (0.148)<br>p: <0.001
**Education - Graduate**	1.175 *** (0.141)<br>p: <0.001	1.126 *** (0.140)<br>p: <0.001	1.208 *** (0.141)<br>p: <0.001
**Religiosity - Religious, actively practicing**	-0.315 *** (0.070)<br>p: <0.001	--	-0.378 *** (0.067)<br>p: <0.001
**Religiosity - Religious, not actively practicing**	-0.135 * (0.066)<br>p: 0.041	--	-0.174 ** (0.065)<br>p: 0.008
**Political Belief - Conservative**	0.034 (0.068)<br>p: 0.611	-0.016 (0.067)<br>p: 0.812	--
**Political Belief - Liberal**	0.259 *** (0.063)<br>p: <0.001	0.295 *** (0.062)<br>p: <0.001	--
n	3790	3790	3790
R^2	0.125	0.120	0.121
Overall *F* statistic	67.595	86.210	86.548
Overall *F p* value	<0.001	<0.001	<0.001

*** $p < 0.001$; ** $p < 0.01$; * $p < 0.05$.

Table S9: Linear regression model results for the Skills GL score by both religiosity and political belief; only political belief; or only religiosity. Entries are the "coefficient (standard error)," and stars indicate statistical significance.

Religious Affiliation and Political Belief

For all three genetic literacy scores, there does not seem to be much information gained by adding religious affiliation to the model that already includes population, education, and political belief or by adding political belief to the model that already includes population, education, and religious affiliation. The absolute changes in R^2 are all less than 1%.
The overall R^2 values are slightly higher for religiosity than for religious affiliation in the models for all three GL scores, but the differences are small.

Familiarity Score

	Full Model	Political Belief Only	Religious Affiliation Only
**Intercept**	4.505 *** (0.114)<br>p: <0.001	4.407 *** (0.110)<br>p: <0.001	4.628 *** (0.112)<br>p: <0.001
**Population - SPARK**	0.364 *** (0.043)<br>p: <0.001	0.374 *** (0.043)<br>p: <0.001	0.397 *** (0.042)<br>p: <0.001
**Education - High School**	0.719 *** (0.114)<br>p: <0.001	0.703 *** (0.114)<br>p: <0.001	0.719 *** (0.114)<br>p: <0.001
**Education - Undergraduate**	0.957 *** (0.118)<br>p: <0.001	0.933 *** (0.118)<br>p: <0.001	0.974 *** (0.118)<br>p: <0.001
**Education - Graduate**	1.159 *** (0.112)<br>p: <0.001	1.132 *** (0.112)<br>p: <0.001	1.192 *** (0.112)<br>p: <0.001
**Religious**	-0.158 *** (0.048)<br>p: <0.001	--	-0.209 *** (0.047)<br>p: <0.001
**Political Belief - Conservative**	0.052 (0.054)<br>p: 0.334	0.031 (0.053)<br>p: 0.561	--
**Political Belief - Liberal**	0.280 *** (0.050)<br>p: <0.001	0.301 *** (0.050)<br>p: <0.001	--
n	3790	3790	3790
R^2	0.086	0.083	0.077
Overall *F* statistic	50.686	57.166	63.579
Overall *F* *p* value	<0.001	<0.001	<0.001

*** $p < 0.001$; ** $p < 0.01$; * $p < 0.05$.

Table S10: Linear regression model results for the Familiarity GL score by both religious affiliation and political belief; only political belief; or only religious affiliation. Entries are the "coefficient (standard error)," and stars indicate statistical significance.

Knowledge Score

	Full Model	Political Belief Only	Religious Affiliation Only
**Intercept**	7.223 *** (0.272)<br>p: <0.001	6.963 *** (0.263)<br>p: <0.001	7.536 *** (0.266)<br>p: <0.001
**Population - SPARK**	2.124 *** (0.102)<br>p: <0.001	2.151 *** (0.102)<br>p: <0.001	2.166 *** (0.101)<br>p: <0.001
**Education - High School**	1.193 *** (0.271)<br>p: <0.001	1.151 *** (0.271)<br>p: <0.001	1.205 *** (0.272)<br>p: <0.001
**Education - Undergraduate**	1.727 *** (0.281)<br>p: <0.001	1.663 *** (0.281)<br>p: <0.001	1.778 *** (0.282)<br>p: <0.001
**Education - Graduate**	2.603 *** (0.267)<br>p: <0.001	2.533 *** (0.267)<br>p: <0.001	2.686 *** (0.268)<br>p: <0.001
**Religious**	-0.418 *** (0.114)<br>p: <0.001	--	-0.479 *** (0.111)<br>p: <0.001
**Political Belief - Conservative**	0.384 ** (0.128)<br>p: 0.003	0.329 ** (0.127)<br>p: 0.010	--
**Political Belief - Liberal**	0.622 *** (0.119)<br>p: <0.001	0.680 *** (0.118)<br>p: <0.001	--
n	3790	3790	3790
R^2	0.181	0.178	0.175
Overall *F* statistic	119.517	136.742	160.643
Overall *F* *p* value	<0.001	<0.001	<0.001

*** $p < 0.001$; ** $p < 0.01$; * $p < 0.05$.

Table S11: Linear regression model results for the Knowledge GL score by both religious affiliation and political belief; only political belief; or only religious affiliation. Entries are the “coefficient (standard error),” and stars indicate statistical significance.

Skills Score

	Full Model	Political Belief Only	Religious Affiliation Only
**Intercept**	1.843 *** (0.143)<br>p: <0.001	1.710 *** (0.138)<br>p: <0.001	1.954 *** (0.140)<br>p: <0.001
**Population - SPARK**	0.870 *** (0.054)<br>p: <0.001	0.884 *** (0.054)<br>p: <0.001	0.906 *** (0.053)<br>p: <0.001
**Education - High School**	0.643 *** (0.143)<br>p: <0.001	0.622 *** (0.143)<br>p: <0.001	0.642 *** (0.143)<br>p: <0.001
**Education - Undergraduate**	0.957 *** (0.148)<br>p: <0.001	0.925 *** (0.148)<br>p: <0.001	0.971 *** (0.148)<br>p: <0.001
**Education - Graduate**	1.162 *** (0.141)<br>p: <0.001	1.126 *** (0.140)<br>p: <0.001	1.192 *** (0.141)<br>p: <0.001
**Religious**	-0.213 *** (0.060)<br>p: <0.001	--	-0.269 *** (0.058)<br>p: <0.001
**Political Belief - Conservative**	0.012 (0.067)<br>p: 0.857	-0.016 (0.067)<br>p: 0.812	--
**Political Belief - Liberal**	0.266 *** (0.063)<br>p: <0.001	0.295 *** (0.062)<br>p: <0.001	--
n	3790	3790	3790
R^2	0.123	0.120	0.118
Overall *F* statistic	75.925	86.210	101.450
Overall *F p* value	<0.001	<0.001	<0.001
*** $p < 0.001$; ** $p < 0.01$; * $p < 0.05$.			

Table S12: Linear regression model results for the Skills GL score by both religious affiliation and political belief; only political belief; or only religious affiliation. Entries are the "coefficient (standard error)," and stars indicate statistical significance.

Interactions with Education

There are no statistically significant interactions between education and religious affiliation, religiosity, or political belief.

Religious Affiliation

Variable	Familiarity Score	Knowledge Score	Skills Score
**Population**	<0.001	<0.001	<0.001
**Education Level**	<0.001	<0.001	<0.001
**Religious Affiliation**	<0.001	<0.001	<0.001
**Education Level*Religious Affiliation**	0.091	0.709	0.102

Table S13: ANOVA results for interaction test between education level and religious affiliation, with each value reporting statistical significance of the interaction.

	Familiarity Score	Knowledge Score	Skills Score
**Intercept**	4.907 *** (0.165)<br>p: <0.001	7.730 *** (0.393)<br>p: <0.001	2.306 *** (0.204)<br>p: <0.001
**Population - SPARK**	0.320 *** (0.041)<br>p: <0.001	1.949 *** (0.099)<br>p: <0.001	0.837 *** (0.051)<br>p: <0.001
**Education - High School**	0.347 * (0.176)<br>p: 0.050	0.848 * (0.421)<br>p: 0.044	0.216 (0.218)<br>p: 0.324
**Education - Undergraduate**	0.710 *** (0.188)<br>p: <0.001	1.518 *** (0.447)<br>p: <0.001	0.673 ** (0.232)<br>p: 0.004
**Education - Graduate**	0.950 *** (0.175)<br>p: <0.001	2.616 *** (0.417)<br>p: <0.001	0.772 *** (0.216)<br>p: <0.001
**Religious**	-0.637 ** (0.213)<br>p: 0.003	-0.746 (0.507)<br>p: 0.141	-0.834 ** (0.263)<br>p: 0.002
**Education - High School*Religious**	0.541 * (0.226)<br>p: 0.017	0.429 (0.540)<br>p: 0.427	0.635 * (0.280)<br>p: 0.024
**Education - Undergraduate*Religious**	0.388 (0.237)<br>p: 0.102	0.302 (0.566)<br>p: 0.594	0.521 (0.294)<br>p: 0.076
**Education - Graduate*Religious**	0.399 (0.223)<br>p: 0.074	0.172 (0.533)<br>p: 0.747	0.661 * (0.277)<br>p: 0.017
n	4057	4057	4057
R^2	0.070	0.156	0.108
Overall *F* statistic	38.068	93.237	60.968
Overall *F* *p* value	<0.001	<0.001	<0.001
*** $p < 0.001$; ** $p < 0.01$; * $p < 0.05$.			

Table S14: Linear regression model results for each GL score by population, education, and interaction between education and religious affiliation. Entries are the “coefficient (standard error),” and stars indicate statistical significance.

Religiosity

Variable	Familiarity Score	Knowledge Score	Skills Score
Population	<0.001	<0.001	<0.001
Education Level	<0.001	<0.001	<0.001
Religiosity	<0.001	<0.001	<0.001
Education Level*Religiosity	0.338	0.950	0.202

Table S15: ANOVA results for interaction test between education level and religiosity, with each value reporting statistical significance of the interaction.

	Familiarity Score	Knowledge Score	Skills Score
**Intercept**	4.911 *** (0.164) p: <0.001	7.736 *** (0.393) p: <0.001	2.311 *** (0.204) p: <0.001
**Population - SPARK**	0.307 *** (0.042) p: <0.001	1.933 *** (0.099) p: <0.001	0.819 *** (0.051) p: <0.001
**Education - High School**	0.349 * (0.176) p: 0.048	0.850 * (0.421) p: 0.043	0.219 (0.218) p: 0.316
**Education - Undergraduate**	0.714 *** (0.187) p: <0.001	1.523 *** (0.447) p: <0.001	0.680 ** (0.232) p: 0.003
**Education - Graduate**	0.954 *** (0.175) p: <0.001	2.621 *** (0.417) p: <0.001	0.778 *** (0.216) p: <0.001
**Religiosity - Religious, not actively practicing**	-0.555 * (0.248) p: 0.025	-0.837 (0.592) p: 0.157	-0.909 ** (0.307) p: 0.003
**Religiosity - Religious, actively practicing**	-0.727 ** (0.256) p: 0.004	-0.645 (0.610) p: 0.291	-0.750 * (0.316) p: 0.018
**Education - High School*Religiosity - Religious, not actively practicing**	0.517 * (0.263) p: 0.049	0.618 (0.627) p: 0.324	0.780 * (0.325) p: 0.016
**Education - Undergraduate*Religiosity - Religious, actively practicing**	0.337 (0.283) p: 0.233	0.057 (0.675) p: 0.933	0.273 (0.350) p: 0.436
**Education - Graduate*Religiosity - Religious, not actively practicing**	0.408 (0.260) p: 0.116	0.385 (0.620) p: 0.535	0.877 ** (0.322) p: 0.006
n	4057	4057	4057
R^2	0.073	0.156	0.111
Overall *F* statistic	26.620	62.504	42.149
Overall *F p* value	<0.001	<0.001	<0.001

*** $p < 0.001$; ** $p < 0.01$; * $p < 0.05$.

Table S16: Linear regression model results for each GL score by population, education, and interaction between education and religiosity. Entries are the "coefficient (standard error)," and stars indicate statistical significance.

Political Belief

Variable	Familiarity Score	Knowledge Score	Skills Score
Population	<0.001	<0.001	<0.001
Education Level	<0.001	<0.001	<0.001
Political Belief	<0.001	<0.001	<0.001
Education Level*Political Belief	0.578	0.743	0.372

Table S17: ANOVA results for interaction test between education level and political belief, with each value reporting statistical significance of the interaction.

	**Familiarity Score**	**Knowledge Score**	**Skills Score**
**Intercept**	4.594 *** (0.161)<br>p: <0.001	7.087 *** (0.383)<br>p: <0.001	1.685 *** (0.201)<br>p: <0.001
**Population - SPARK**	0.373 *** (0.043)<br>p: <0.001	2.152 *** (0.102)<br>p: <0.001	0.884 *** (0.054)<br>p: <0.001
**Education - High School**	0.496 ** (0.171)<br>p: 0.004	1.035 * (0.407)<br>p: 0.011	0.653 ** (0.214)<br>p: 0.002
**Education - Undergraduate**	0.817 *** (0.180)<br>p: <0.001	1.704 *** (0.430)<br>p: <0.001	0.781 *** (0.226)<br>p: <0.001
**Education - Graduate**	0.910 *** (0.170)<br>p: <0.001	2.310 *** (0.405)<br>p: <0.001	1.225 *** (0.213)<br>p: <0.001
**Political Belief - Conservative**	-0.275 (0.272)<br>p: 0.312	0.517 (0.648)<br>p: 0.425	-0.028 (0.341)<br>p: 0.935
**Political Belief - Liberal**	-0.061 (0.249)<br>p: 0.808	0.141 (0.594)<br>p: 0.812	0.383 (0.313)<br>p: 0.220
**Education - High School*Political Belief - Conservative**	0.325 (0.287)<br>p: 0.258	-0.271 (0.684)<br>p: 0.692	-0.014 (0.360)<br>p: 0.968
**Education - Undergraduate*Political Belief - Liberal**	0.271 (0.274)<br>p: 0.322	0.269 (0.654)<br>p: 0.681	0.206 (0.344)<br>p: 0.549
**Education - Graduate*Political Belief - Conservative**	0.376 (0.284)<br>p: 0.186	-0.036 (0.676)<br>p: 0.958	-0.063 (0.356)<br>p: 0.860
n	3790	3790	3790
R^2	0.084	0.179	0.122
Overall *F* statistic	28.968	68.618	43.650
Overall *F p* value	<0.001	<0.001	<0.001

*** $p < 0.001$; ** $p < 0.01$; * $p < 0.05$.

Table S18: Linear regression model results for each GL score by population, education, and interaction between education and political belief. Entries are the "coefficient (standard error)," and stars indicate statistical significance.
